# A bioluminescent and homogeneous SARS-CoV-2 spike RBD and hACE2 interaction assay for antiviral screening and monitoring patient neutralizing antibody levels

**DOI:** 10.1038/s41598-021-97330-3

**Published:** 2021-09-16

**Authors:** Juliano Alves, Laurie Engel, Renata de Vasconcelos Cabral, Eduardo L. Rodrigues, Liane de Jesus Ribeiro, Luiza M. Higa, Orlando da Costa Ferreira Júnior, Terezinha Marta P. P. Castiñeiras, Isabela de Carvalho Leitão, Amilcar Tanuri, Said A. Goueli, Hicham Zegzouti

**Affiliations:** 1 R&D Department, Promega Corporation, Madison, WI USA; 2 Laboratório de Virologia Molecular, Departamento de Genética, Instituto de Biologia, Universidade Federal Do Rio de Janeiro, Rio de Janeiro, Brazil; 3 Promega Biotecnologia Do Brasil, São Paulo, Brazil; 4 Departamento de Doenças Infecciosas E Parasitárias, Faculdade de Medicina, Universidade Federal Do Rio de Janeiro, Rio de Janeiro, Brazil; 5 Instituto de Biofísica Carlos Chagas Filho, Universidade Federal Do Rio de Janeiro, Rio de Janeiro, Brazil; 6 Department of Pathology and Laboratory Medicine, University of Wisconsin School of Medicine and Public Health, Madison, WI USA

**Keywords:** Infectious diseases, Biological techniques, Immunology

## Abstract

Here we describe a homogeneous bioluminescent immunoassay based on the interaction between Fc-tagged SARS-CoV-2 Spike RBD and human ACE2, and its detection by secondary antibodies labeled with NanoLuc luciferase fragments LgBit and SmBit. The assay utility for the discovery of novel inhibitors was demonstrated with a panel of anti-RBD antibodies, ACE2-derived miniproteins and soluble ACE2. Studying the effect of RBD mutations on ACE2 binding showed that the N501Y mutation increased RBD apparent affinity toward ACE2 tenfold that resulted in escaping inhibition by some anti-RBD antibodies. In contrast, while E484K mutation did not highly change the binding affinity, it still escaped antibody inhibition likely due to changes in the epitope recognized by the antibody. Also, neutralizing antibodies (NAbs) from COVID-19 positive samples from two distinct regions (USA and Brazil) were successfully detected and the results further suggest the persistence of NAbs for at least 6 months post symptom onset. Finally, sera from vaccinated individuals were tested for NAbs and showed varying neutralizing activity after first and second doses, suggesting the assay can be used to assess immunity of vaccinated populations. Our results demonstrate the broad utility and ease of use of this methodology both for drug discovery and clinical research applications.

## Introduction

The coronavirus disease 2019 (COVID-19) caused by the severe acute respiratory syndrome coronavirus 2 (SARS-CoV-2), was initially detected in Wuhan, China in December 2019, and it has now become a worldwide pandemic. As of April 2021, it has infected 137,866,311 people and claimed 2,965,707 lives^1^. Worldwide collaborations across governments, academia and private sectors resulted in the development of an unprecedented number of diagnostic assays, drugs, and vaccine candidates against COVID-19, which are now approved or expected to be approved for use in record time.

SARS-CoV-2 is a member of the genus Betacoronavirus, which also includes other viruses such as SARS-CoV and MERS-CoV^2^ that can cause respiratory illness. SARS-CoV-2 virus enters target cells (e.g.: ciliated bronchial epithelial cells, endothelial cells, and type I and II pneumocytes) after binding of its surface Spike protein (S) to the Angiotensin Converting Enzyme 2 (ACE2) receptor^3,4^. The Spike glycoprotein is a homotrimer composed of two subunits: the S1 subunit comprises the Receptor Binding Domain (RBD), which first recognizes the ACE2 receptor, and the S2 subunit, which then undergoes a conformational change promoting the cellular internalization of the viral particle^4,5^.

The importance of the Spike protein for viral internalization is highlighted by efforts to identify molecules that could interfere with its binding to ACE2. Through in silico docking studies, Benítez-Cardoza and Vique-Sánchez identified 20 commercially available compounds with a predicted favorable toxicity profile that might inhibit the Spike:ACE2 interaction^6^. In addition, Choudhary et al. reported on the use of in silico high throughput screening to investigate the FDA LOPAC library, which led to the identification of potential candidates for drug repurposing^7^. Huang et al. described the computational design of 31-mer peptidic scaffolds by linking fragments derived from ACE2^8^, and Cao et al. reported the design and inhibitory activities of peptides designed around the ACE2 helix that interacts with RBD^9^. Also, Wu et al. described the isolation of patient-derived neutralizing antibodies that block the interaction between RBD and ACE2^10^, demonstrating the feasibility of antibody-based therapeutics, and Lei et al. described the ability of soluble ACE2-Fc to impair viral entry^11^.

Since the Spike RBD comprises one of the immunodominant regions of SARS-CoV-2, several methods that detect the presence of neutralizing antibodies (NAbs) against RBD have been devised. Two cell-based methods are currently used for detection and titration of anti-SARS-CoV-2 neutralizing antibodies: the Plaque Reduction Neutralization Test (PRNT) and the Viral Neutralization Test (VNT). Both cell-based methods assess antibody titers in patient samples through serial dilutions of serum or plasma samples pre-incubated either with viable SARS-CoV-2 virus (PRNT), or with pseudotyped viral particles expressing the Spike protein (VNT)^3,12,13^. Alternatively, in vitro biochemical assays have also been developed for this purpose. Tan et al. recently described the development of SARS-CoV-2 Spike RBD-streptavidin:ACE2 ELISA-based assay capable of detecting neutralizing antibodies in patient serum. The method showed good correlation with both conventional virus neutralization test (cVNT) and pseudovirus-based VNT (pVNT), suggesting that it could be used as a surrogate assay^14^. Azad et al. described the development of a Nanoluc luciferase complementation reporter assay using secreted media and cell-free extracts containing NanoBit-labeled SARS-CoV-2 Spike RBD and hACE2. The authors showed that both cell-free extracts and secreted media could be used for screening of monoclonal antibodies, detection of Spike RBD-neutralizing antibodies in patient serum as well as inhibition of Spike RBD:ACE2 interaction^15^. While these methods enable the detection and characterization of patient-derived neutralizing antibodies, they suffer from several limitations. For instance, cell-based assays like PRNT and VNT require the use of either live virus or virus-like particles to detect NAbs in patient derived samples. Consequently, these assays must be performed in BSL3 and BSL2 environments, respectively. ELISA for monitoring RBD:ACE2 interactions are labor intensive, time consuming and low throughput. Therefore, a simple assay that monitors the interaction of SARS-CoV-2 Spike RBD and human ACE2 could be a useful tool both for high throughput screening as well as for the screening and characterization of clinical specimens that may contain virus neutralizing antibodies.

In this study, we describe the development of a no-wash, add-and-read bioluminescence-based immunoassay that relies on detection of the recombinant SARS-CoV-2 Spike RBD and hACE2 protein–protein interaction (PPI) using antibodies labeled with NanoBiT subunits (Lumit anti-mouse Ab-LgBiT and Lumit anti-rabbit Ab-SmBiT)^16^. This plate-based assay can be performed in 2 h with good signal-to-background values and the luminescent signal is stable for up to 2 h. The approach was validated by testing the effect of anti-SARS-CoV-2 Spike RBD monoclonal antibodies and soluble hACE2 on the RBD:ACE2 PPI. Most importantly the assay enabled the identification of PPI inhibitors and the presence of NAbs in patient plasma and serum samples. Finally, using this assay in a competition mode to study the effect of new RBD mutant variants on the binding to ACE2 showed a broad range of affinities correlating with known levels of infectivity of these virus variants and their escape of inhibition by anti-RBD antibodies.

We believe this approach, applied here to SARS-CoV-2 or to other viral diseases, could have many applications such as enabling the screening of novel inhibitors, studying population-wide herd immunity, tracking potential viral reservoirs in the wild, and assisting the screening of convalescent plasma with adequate antibody titers for transfusion into critically ill patients.

## Results

### Development of a homogeneous bioluminescent SARS-CoV-2 Spike RBD:ACE2 Immunoassay

**Figure 1:**
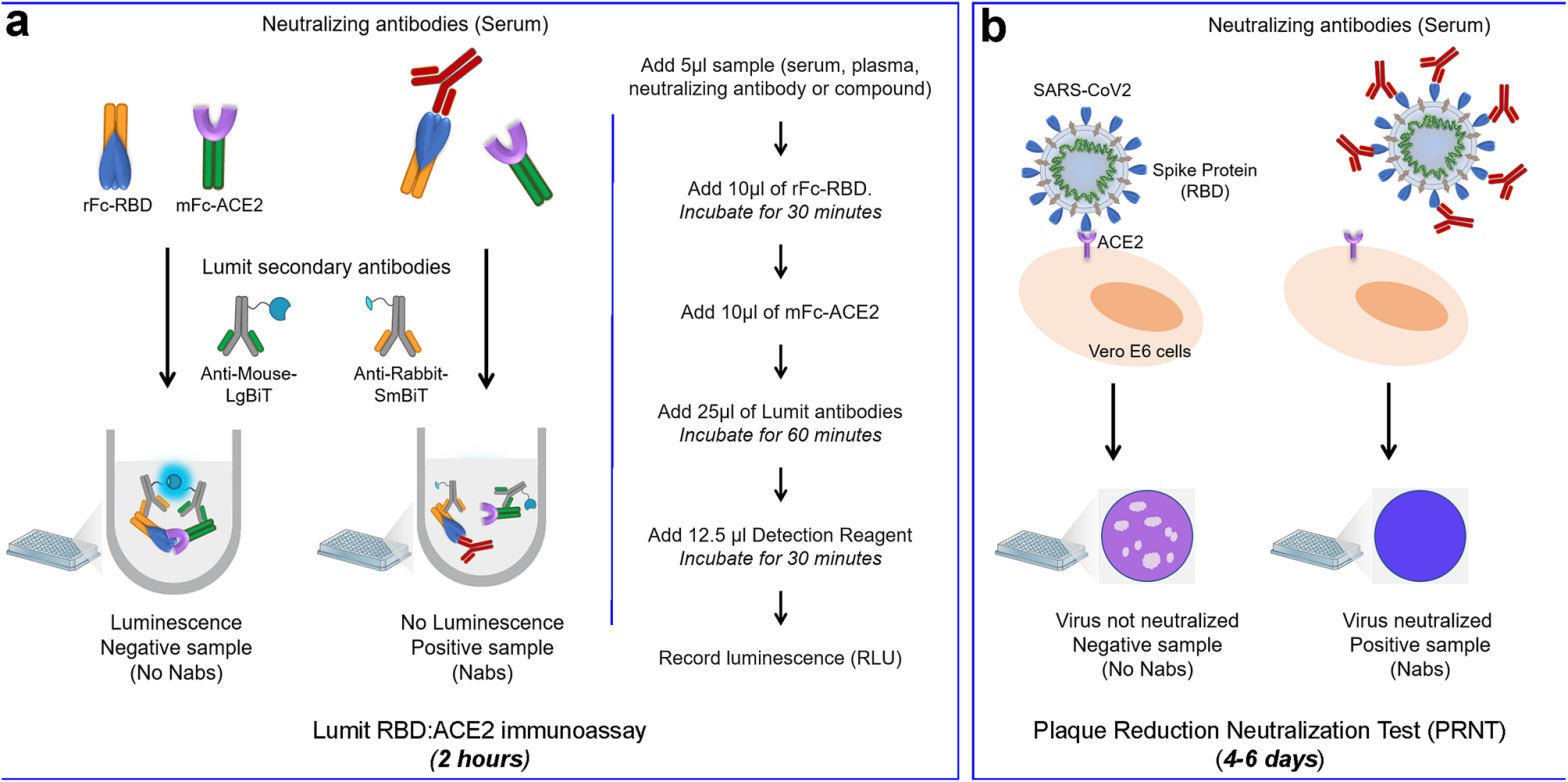
Schematic representation of the Lumit SARS-CoV-2 Spike RBD:ACE2 Immunoassay and comparison with PRNT assay. (**a**) Lumit SARS-CoV-2 Spike RBD:ACE2 Immunoassay detects the interaction between the rabbit Fc-tagged RBD fragment of SARS-CoV-2 Spike protein and the soluble mouse Fc-tagged ACE2 receptor through binding of LgBit/SmBit-conjugated Lumit secondary antibodies. Lumit secondary antibody recognition of RBD:ACE2 interaction leads to NanoLuc fragments complementation and bioluminescent signal. Lumit SARS-Cov-2 Spike RBD:ACE2 Immunoassay protocol. Sample (i.e.: antibody, inhibitor, serum, plasma, etc.) is co-incubated with Spike RBD-rabbit Fc for 30 min at room temperature. hACE2-mouse Fc and Lumit secondary antibodies are added to the samples and the assay reactions are incubated at RT for 60 min. Bioluminescence is recorded in a luminometer after the addition of Detection Reagent and incubation for 30 min at room temperature. (**b**) Plaque Reduction Neutralization Test. Serially diluted patient samples are incubated with viable SARS-CoV-2 viral particles before dispensing onto VeroE6 cells. Formation of plaques due to cytopathic effect are inhibited if neutralizing antibodies are present, and the titer is determined by the serum dilution required to inhibit plaque formation by 90% or 50% (i.e.: PRNT90 or PRNT50, respectively).

**Figure 2:**
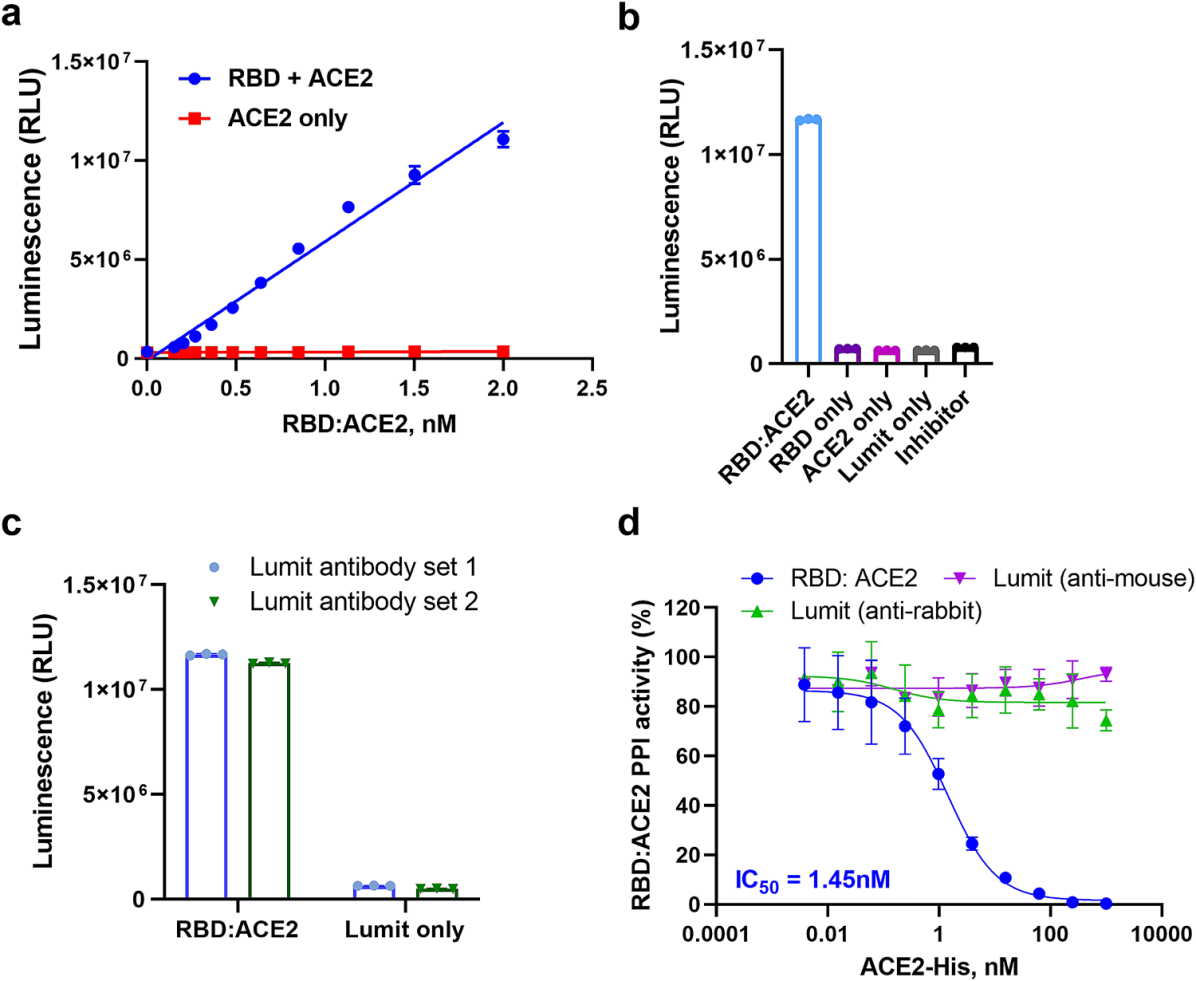
Validation of Lumit SARS-CoV-2 Spike RBD:ACE2 Immunoassay. (**a**) Assay linearity of Lumit SARS-CoV-2 Spike RBD:ACE2 Immunoassay. SARS-CoV-2 Spike RBD:ACE2 titration was performed in 1× Buffer. (**b**) Spike RBD:ACE2-mediated antibody proximity leads to NanoLuc complementation and bioluminescent signal. The absence of either assay component results in loss of signal. Bioluminescence is dependent on the RBD:ACE2 interaction as evidenced by inhibition with anti-Spike RBD antibody. (**c**) SARS-CoV-2 Spike RBD:ACE2 detection is not affected by the orientation of NanoLuc reporter fragment in secondary antibodies. Set 1: Lumit Anti-Mouse Ab-LgBiT/Lumit Anti-Rabbit Ab-SmBiT. Set 2: Lumit Anti-Mouse Ab-SmBiT/Lumit Anti-Rabbit Ab-LgBiT. (**d**) RBD:ACE2 interaction is inhibited by soluble ACE2-His. As control, ACE2-His dilutions were incubated with a luminescence-generating reaction that contained either 1.5 nM RBD-rabbit Fc and Lumit Anti-Rabbit (SmBit/LgBit) antibodies, or 1.5 nM ACE2-mouse Fc and Lumit Anti-Mouse (SmBit/LgBit) antibodies in 1× Lumit immunoassay reaction buffer, and assay was performed as described above. Results are presented as means ± S.E.M. (n = 3 technical replicates, the data are representative of two or more experiments).

A screening assay that monitors the interaction of SARS-CoV-2 Spike RBD and human Angiotensin Converting Enzyme 2 (hACE2) would be desirable for allowing both the discovery of inhibitory molecules—e.g., small molecules, monoclonal antibodies, biologics—capable of interfering with their interaction, as well as for identification of circulating antibodies in seroconverted individuals. We therefore sought to develop a homogeneous, no-wash bioluminescent assay that monitors the protein–protein interaction between soluble SARS-CoV-2 Spike RBD and hACE2 using the Lumit immunoassay system previously developed by our team^16^. The Lumit immunoassay combines immunodetection and NanoLuc Binary Technology (NanoBiT), a two-subunit system based on NanoLuc luciferase that enables the detection of protein–protein interaction (PPI) when the subunits are either expressed as recombinant fusions or chemically conjugated to target proteins of interest. In the Lumit Immunoassay, NanoBiT subunits (LgBiT and SmBiT) are conjugated to a pair of antibodies that recognize two different target epitopes. If the two epitopes are in close proximity, binding of the NanoBiT-labeled antibodies (Lumit antibodies) to their corresponding epitopes brings NanoBiT subunits into proximity, reconstituting an active NanoLuc luciferase, which then generates light in proportion to the amount of target. The luminescent signal generated is determined using a simple luminometer. For this study, to create a biochemical Lumit RBD:ACE2 PPI assay, we used Lumit antibodies that recognize tags on recombinant RBD and ACE2 proteins, thus preventing any interference of the antibodies with the PPI interface. Previously, our group created Lumit secondary antibodies against different IgG species—e.g., anti-rabbit and anti-mouse -that were used in combination with non-labelled primary antibodies in immunoassays for detection of multiple intracellular targets^16^. As these Lumit secondary antibodies can also bind to their corresponding immunoglobulin Fc species fragments, we began evaluating recombinant SARS-CoV-2 Spike RBD and hACE2 constructs with Fc tags that could be paired with available Lumit secondary antibodies. We identified two constructs, SARS-CoV-2 Spike RBD-rabbit Fc and hACE2-mouse Fc that could be paired with Lumit anti-rabbit and anti-mouse antibodies. In principle, interaction of RBD-Fc and ACE2-Fc in solution along with binding of the Lumit antibodies to their corresponding Fc would bring NanoBiT subunits into proximity to generate light in proportion to the level of PPI (Fig. 1a). Both Spike RBD-Fc and hACE2-Fc proteins were serially diluted from 2 to 0.2 nM, and the addition of both proteins to the assay reaction mixture containing Lumit antibodies led to the antibody mediated NanoLuc complementation as evidenced by the increase in bioluminescence. Also, the results indicate the assay is linear up to 2 nM for both proteins with significant signal-to-background values (Fig. 2a and supplementary Fig. 1b). In contrast, light output dependent on either Spike RBD-Fc alone or hACE2-Fc alone was not observed (Fig. 2a, b). Also, Lumit antibodies alone were not able to promote NanoLuc luciferase complementation, as indicated by the low background observed (Fig. 2b).

To demonstrate that the bioluminescent signal was due to a Spike RBD and hACE2 interaction, Spike RBD-Fc was co-incubated with an anti-Spike RBD monoclonal antibody prior to the addition of hACE2-Fc and the Lumit antibodies. The anti-RBD antibody blocked Spike RBD:ACE2 interaction as indicated by the reduction of bioluminescence to background levels (Fig. 2b). Therefore, our results demonstrate that the bioluminescence observed was due to NanoLuc luciferase complementation mediated by the interaction between Spike RBD and human ACE2.

Given that NanoBiT subunit configuration in Lumit secondary antibodies could play a role in the luminescence level generated during the detection of Spike RBD:ACE2 interaction, we evaluated which pair (Lumit anti-rabbit Ab-SmBiT/Lumit anti-mouse Ab-LgBiT; or Lumit anti-rabbit Ab-LgBiT/Lumit anti-mouse Ab-SmBiT) would have the most favorable performance. Both Lumit pairs were tested in the SARS-CoV-2 Spike RBD-rabbit Fc and hACE2-mouse Fc PPI reaction. The results indicate that either pair had similar performance toward detecting Spike RBD:ACE2 interaction (Fig. 2c). To evaluate the assay format and signal stability, both proteins were tested at a final concentration of 1.5 nM and the assay protocol was established (Fig. 1a). The assay reaction can also be miniaturized from a 96-well regular plate to a low volume 384-well assay plate with similar performance (Supplementary Fig. 1a, b), and the bioluminescence signal generated in all formats tested was stable for the course of 2 h at room temperature (Supplementary Fig. 1c).

To validate the assay as a tool for screening novel inhibitory molecules, Spike RBD-Fc was co-incubated with soluble his-tagged hACE2 prior to the addition of hACE2-Fc and the Lumit antibodies. The soluble his-tagged hACE2 competed with hACE2-Fc and disrupted the Spike RBD:ACE2 interaction in a dose-dependent manner (Fig. 2d), indicating that the methodology could be used for the discovery of Spike RBD:ACE2 PPI inhibitors. An additional benefit to the direct detection of Fc-tagged proteins using Lumit secondary antibodies versus using either paired primary antibodies or primary antibodies labelled with NanoBiT subunits, is the avoidance of potential false positives (i.e.: assay reagent and antibodies competing for the same epitopes on RBD or ACE2, leading to assay interference). We observed this issue with an initial assay design, which utilized a rabbit anti-SARS-CoV-2 Spike RBD primary antibody paired with Lumit anti-rabbit antibody (data not shown).

### Screening and characterization of RBD:ACE2 PPI inhibitors

Since the Spike RBD:ACE2 Immunoassay was successfully demonstrated to detect inhibition of Spike RBD and hACE2 interaction by using both a soluble receptor and a monoclonal antibody, we decided to screen several commercially available monoclonal antibodies (Table 1). Fifteen human anti-SARS-CoV and anti-SARS-CoV-2 monoclonal antibodies and antibody fragments were screened by incubating with RBD-Fc for 30 min before the addition of hACE2-Fc and the remaining Lumit components. We observed different inhibitory activities across the antibodies tested. Antibodies originally isolated during the SARS outbreak in 2002–2003 (CR3022) cross-reacted with SARS-CoV-2 antigens but were unable to completely abrogate the SARS-CoV2 Spike RBD:ACE2 interaction, whereas anti-SARS-CoV-2 Spike antibodies showed strong inhibition. We were unable to detect any RBD:ACE2 inhibition mediated by anti-SARS-CoV-2 nucleocapsid (NP) antibody, demonstrating that disruption of RBD:ACE2 interaction was specific (Fig. 3a). The positive hits were further characterized in dose–response experiments. Our results demonstrate that the antibodies could be divided into three different groups based on their potencies: 1—antibodies with strong inhibition as evidenced by their low nanomolar IC_50_ values; 2—antibodies with partial inhibition (i.e.: antibodies that reach ~ 50% inhibition maximum); and 3—antibodies with weak or no inhibition (Fig. 3b). The antibodies did not interfere with NanoBit luciferase activity when different concentrations were incubated either with RBD-Fc and Lumit Anti-Rabbit antibodies, or with hACE2-Fc and Lumit Anti-mouse antibodies (data not shown).

**Table 1:** List of anti-SARS-CoV-2 Spike protein antibodies used in this study.

Antibody #	Antibody description	Company	Catalog #
1	Anti-SARS-CoV-2 Spike RBD Chimeric mAb	Sino Biological	40150-D001
2	Anti-SARS-CoV-2 Spike RBD Chimeric mAb	Sino Biological	40150-D002
3	Human anti-SARS-CoV-2 Spike RBD	Active Motif	91349
4	Human anti-SARS-CoV-2 Spike RBD	Active Motif	91361
5	Human anti-SARS-CoV-2 Spike RBD mAb IgG1	Biolegend	938502
6	Human anti-SARS-CoV-2 Spike RBD mAb IgG1	Biolegend	938602
7	Human anti-SARS-CoV Spike IgG1 [CR3022]	Absolute Antibody	Ab01680-10
8	Human anti-SARS-CoV Spike hIgG1 fc silent [CR3022]	Absolute Antibody	Ab01680-10.3
9	Human anti-SARS-CoV Spike hFab [CR3022]	Absolute Antibody	Ab01680-10.6
10	Human anti-SARS-CoV Spike hF(ab)2 [CR3022]	Absolute Antibody	Ab01680-10.7
11	Human anti-SARS-CoV Spike IgG2 [CR3022]	Absolute Antibody	Ab01680-11.0
12	Human anti-SARS-CoV Spike IgG3 [CR3022]	Absolute Antibody	Ab01680-12.1
13	Human anti-SARS-CoV Spike IgG4 [CR3022]	Absolute Antibody	Ab01680-13.12
14	Human anti-SARS-CoV Spike IgM [CR3022]	Absolute Antibody	Ab01680-15
15	Human anti-SARS-CoV-2 Spike RBD mAb IgG1	Acro Biosystems	SAD-S35
16	Human anti-SARS-CoV-2 Nucleocapsid Chimeric hMab	Invitrogen	MA5-35941

**﻿Figure 3:**
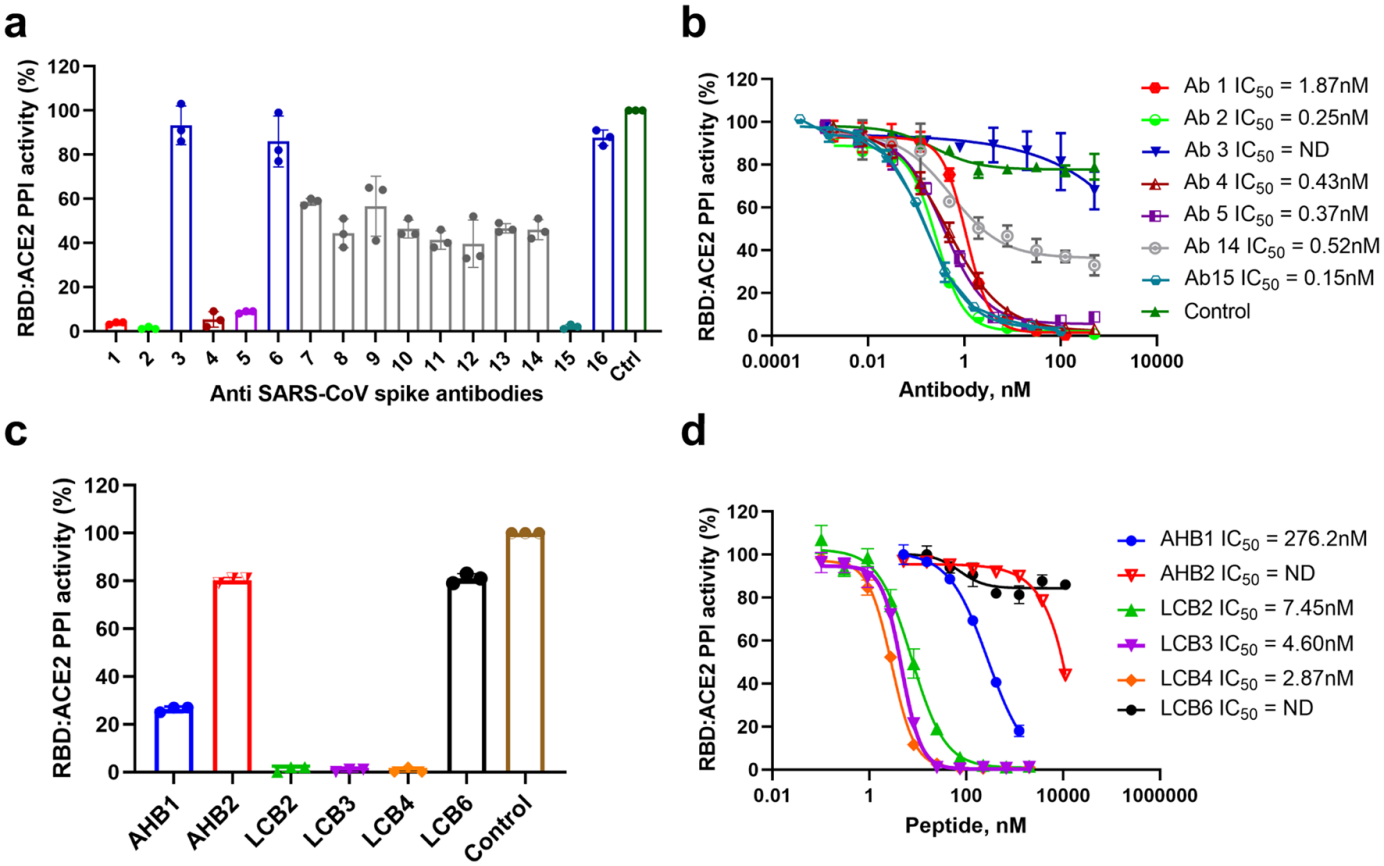
Discovery and characterization of Spike RBD:ACE2 inhibitory molecules. (**a**) Antibody screening. Anti-SARS-CoV-1 and 2 Spike antibodies were tested at 55 nM. Anti-SARS-CoV-2 Nucleocapsid (NP) antibody (#16) was used as a control for specificity and the absence of antibody (Ctrl) was used for 100% activity. (**b**) Antibody dose response experiments. Monoclonal antibodies were serially diluted and incubated with SARS-CoV-2 Spike RBD-rabbit Fc for 30 min prior to the addition of hACE2-mouse Fc and Lumit antibodies. (**c**) Inhibitory peptides were tested at 1 µM for their ability to disrupt the RBD:ACE2 interaction and the absence of peptide (Ctrl) was used for 100% activity. (**d**) Peptides were serially diluted and incubated with SARS-CoV-2 Spike RBD-rabbit Fc for 30 min prior to the addition of hACE2-mouse Fc and Lumit antibodies. The antibodies and peptides in b and d were tested with either RBD-Fc and Lumit Anti-Rabbit antibodies, or with hACE2-Fc and Lumit Anti-mouse antibodies and showed no interference with NanoBit luciferase activity. Results are presented as means ± S.E.M. (n = 3 technical replicates, the data are representative of two or more experiments).

We also evaluated the effect of hACE2-mimmetic peptides recently reported to inhibit the Spike RBD:ACE2 interaction and prevent SARS-CoV-2 infection of mammalian Vero E6 cells^9^ (Supplementary Table 1). These miniproteins were co-incubated with RBD-Fc at a final concentration of 1 µM for 30 min prior to the addition of hACE2-Fc and the Lumit secondary antibodies. Four miniproteins (AHB1, LCB2-4) were capable of inhibiting Spike RBD:ACE2 interaction while two (AHB2 and LCB6) showed weak or no inhibition (Fig. 3c). Three miniproteins (LCB2-4) inhibited Spike RBD:ACE2 interaction in the low nanomolar range, as evidenced by dose response experiments, while two (AHB1-2) inhibited the interaction in the micromolar range (Fig. 3d). The inhibition is specific to the disruption of Spike RBD and hACE2 since these peptides did not inhibit luminescence generated by the control NanoBiT luciferase (SmBiT and LgBiT of anti-Rabbit antibodies bound to RBD-Fc or SmBiT and LgBiT of anti-Mouse antibodies bound to ACE2-Fc) (data no shown).

In summary, our results demonstrate that the Spike RBD:ACE2 immunoassay could potentially be used for the screening of larger antibody libraries, as well as inhibitory peptides for the discovery of therapeutic candidates that could impair SARS-CoV-2 viral entry through Spike RBD:ACE2 interaction inhibition.

### Characterization of SARS-CoV-2 Spike RBD mutations

**Figure 4:**
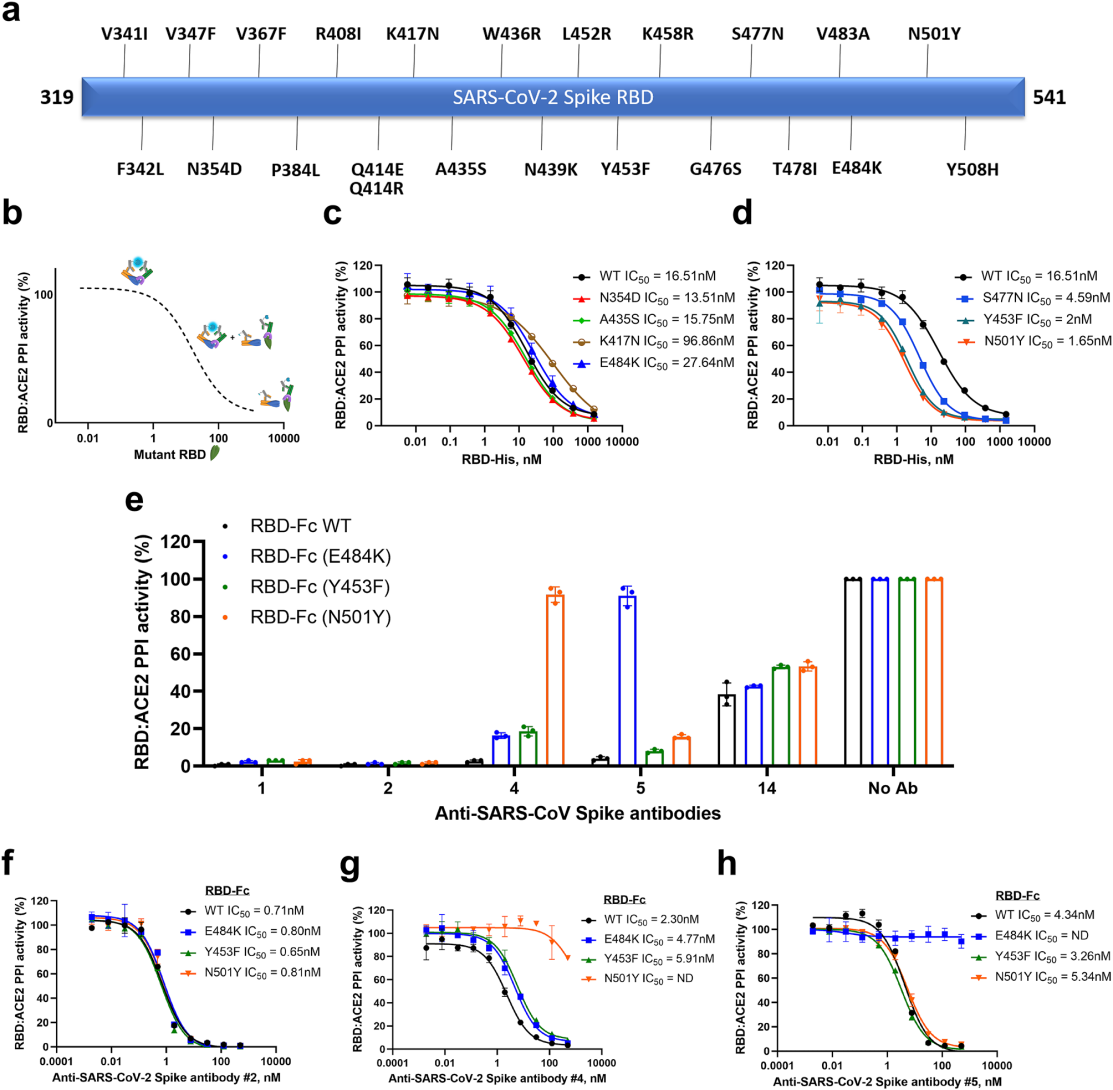
Effect of RBD mutations on Spike RBD:ACE2 binding. (**a**) Schematic diagram showing SARS-CoV-2 Spike RBD mutations evaluated in this study. (**b**) Rationale of the competition assay used for evaluation of RBD mutations. RBD-His mutants were evaluated in dose response experiments, and IC_50_ values generated were compared with wild type RBD-His. (**c**, **d**) RBD-His WT and mutants were serially diluted and incubated with hACE2-mouse Fc for 30 min prior to the addition of RBD-rabbit Fc and Lumit antibodies. (**e**) Antibody screening using wild type RBD-Fc and variants containing the E484K, Y453F or N501Y mutations. (**f**–**h**) Antibody titrations with RBD-Fc WT and mutants. Results are presented as means ± S.E.M. (n = 3 technical replicates, the data are representative of two or more experiments).

Recent reports described the appearance of SARS-CoV-2 variants harboring mutations within the Spike protein sequence, of which some are associated with increased resistance to mAbs or infectivity^17–19^. We decided to evaluate the contribution of mutations within Spike RBD that could influence its interaction with human ACE2 (Fig. 4a). We set up the Spike RBD:ACE2 immunoassay as a competition assay comparing the effect of recombinant wild type RBD protein and its variants harboring different mutations on the displacement of RBD-Fc from hACE2-Fc and inhibition of the PPI. Briefly, if a mutation would increase affinity of RBD toward hACE2, the recombinant RBD mutant would displace RBD-Fc more efficiently in the competition assay than the wild type RBD, thus decreasing the IC_50_ value for the mutant RBD compared to wild type (Fig. 4b). Conversely, if a mutation would reduce RBD affinity, higher concentrations of the mutant would be required to displace wild type RBD-Fc in the assay. Thus, his-tagged SARS-CoV-2 Spike RBD wild type and mutants were serially diluted and pre-incubated with hACE2-Fc for 30 min prior to the addition of RBD-Fc and Lumit antibodies, and their respective IC_50_ values were determined (Supplementary Table 2). The majority of RBD mutations had IC_50_ values comparable to wild type RBD, suggesting that they do not affect RBD interaction with hACE2 (Fig. 4c). However, RBD mutants S477N, Y453F and N501Y showed higher apparent affinity toward hACE2 as evidenced by fourfold, eightfold, and tenfold reductions in their respective IC_50_ values compared to wild type RBD (Fig. 4d and Supplementary Table 2). The E484K mutation that is common in many of the virus variants showed a twofold decrease in affinity to hACE2. It is noteworthy that the N501Y mutation has been linked to increased viral infectivity in BALB/c mice^20^, and it has been identified in SARS-CoV-2 variants isolated in UK, South Africa and Brazil. Furthermore, these mutant variants have been reported to be less responsive to recently developed vaccines against SARS-CoV-2^18,19,21^. However, antibody cocktails could minimize the resistance associated with emerging mutations^22^.

These results prompted us to investigate the effect of mutations E484K, Y453F and N501Y on the binding of neutralizing antibodies described in Fig. 3a, b. RBD-Fc with these mutations were generated and tested for their light generation in the Lumit immunoassay. Their PPI activity with ACE2-Fc generated a specific luminescence and signal window like the wild type RBD-Fc (data not shown). The RBD-Fc mutant assays were then used in antibody testing experiments. The constructs were differentially recognized by the antibodies tested. For instance, while antibodies 1 and 2 showed strong inhibition against all RBD-Fc forms, antibody 4 showed reduced inhibition against RBD-Fc N501Y. Antibody 5 displayed reduced binding to RBD-Fc E484K, while antibody 14 showed intermediate binding toward all RBD-Fc tested (Fig. 4e).

Next, antibodies 2, 4, and 5 were further characterized in dose–response experiments against RBD-Fc mutants to investigate the extent of inhibition escape by these mutants. As previously observed, antibody 2 showed similar inhibitory activity against all four RBD-Fc forms tested (Fig. 4f). The E484K mutation impaired inhibition by antibody 5 (Fig. 4h), whereas RBD-Fc N501Y showed no inhibitory activity by antibody 4 (Fig. 4g). These results suggest that the use of either pan-inhibitory monoclonal antibodies or a combination of antibodies targeting specific mutations could be a feasible approach to treat individuals affected by SARS-CoV-2 variants.

### Effect of patient-derived samples on SARS-CoV-2 Spike RBD:ACE2 interaction

Convalescent plasma from recovered patients containing neutralizing antibodies have efficacy for the treatment of hospitalized individuals affected by severe COVID-19^23^. There are currently two cell-based methods used to characterize anti-SARS-CoV-2 neutralizing antibodies from patient samples. The Plaque Reduction Neutralization Assay (PRNT) consists of pre-incubation of viable SARS-CoV-2 viral particles with serially diluted patient samples, before co-incubation with VeroE6 cells to assess the level of infectivity of the virus^12^. Antibody titers are defined by the serum/plasma dilution that corresponds to 50% (PRNT50) or 90% (PRNT90) inhibition of plaque formation due to virus-mediated cytopathic effects after several days (Fig. 1b). The Viral Neutralization Test (VNT) utilizes luciferase-encoding pseudotyped viral particles—i.e., lentivirus that expresses the Spike protein on the surface and luciferase—and like PRNT, mixtures of viral particles and serially diluted patient samples are co-incubated with human ACE2-overexpressing cell lines (i.e.: HEK293T). Cells are lysed after 48 h, and the resulting luciferase activity is used to determine ND_50_ values^3,13^. An in vitro SARS-CoV-2 Spike RBD-streptavidin:ACE2 ELISA-based assay capable of detecting patient-derived neutralizing antibodies was also developed and demonstrated to have good correlation with both cVNT and pVNT assays^14^.

The detection of circulating neutralizing antibodies could be useful in several ways, such as the screening of convalescent plasma for therapy, immunization effectiveness, population wide exposure and herd immunity, as well as the identification of possible reservoirs in the wild^14^. Since neutralization can be inferred by the disruption of the SARS-CoV-2 Spike RBD:hACE2 interaction, the detection of circulating antibodies in plasma or serum in an in vitro biochemical assay format could streamline the identification of positive samples. Unlike heterogeneous assays such as ELISA which include washing steps to eliminate nonspecific effects, homogeneous assays such as Lumit could be prone to assay matrix interference such as serum. To assess the compatibility of Lumit assay with serum samples, we evaluated the Lumit method using serially diluted negative control serum. We observed a decrease in RLU values with increasing serum concentrations, suggesting a concentration-dependent inhibition of NanoBiT enzyme complementation/activity by the serum matrix (Supplementary Fig. 2a). To minimize interference and define the optimal serum amount to be used in the Lumit assay, we evaluated a patient cohort from Madison, WI comprising 41 COVID-19 PCR positive (true positives) and 43 PCR negative pre-pandemic serum samples at different serum concentrations. As expected, bioluminescence values increased as the serum concentration decreases in both groups. We noticed that serum concentrations higher than 5% (1:20 dilution) superficially increased the neutralization values resulting in an increase in false positives and lowering the specificity of the assay. In contrast, serum concentrations lower than 5% decreased the neutralization values and affected our ability to detect the presence of neutralizing antibodies in patient samples with apparent low antibody titers, thus creating false negatives and decreasing the assay sensitivity. An example of serum effects is shown with a comparison of 4% and 5% serum (Supplementary Fig. 2b, c). Therefore, we selected 5% serum as the final concentration used in the assay for the follow-up experiments.

### COVID-19 positive patient samples inhibit SARS-CoV-2 Spike RBD:ACE2 interaction

**Figure 5:**
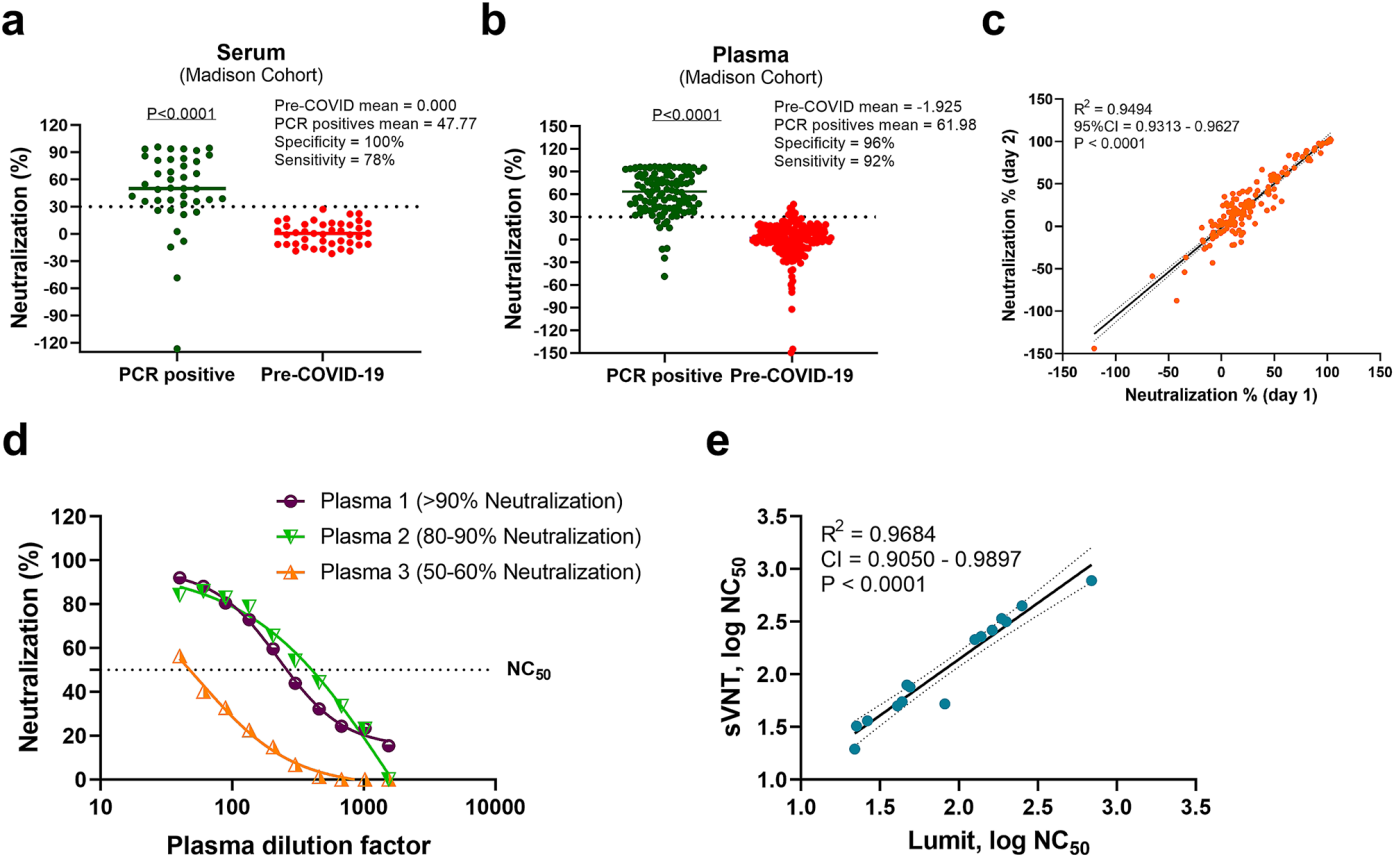
Testing patient derived samples with Lumit SARS-CoV-2 Spike RBD:ACE2 Immunoassay. (**a**) Patient-derived serum samples from 41 COVID-19 positive and 43 negative pre-pandemic samples were tested using Lumit SARS-CoV-2 Spike RBD:ACE2 Immunoassay at 1/20 (5%) final serum dilution. Serum samples were pre-incubated with RBD-Fc for 30 min prior to the addition of the other assay components. (**b**) COVID-19 positive (n = 116) and negative pre-pandemic (n = 202) plasma samples were tested at 1/20 final plasma dilution. A cutoff value of 30% was selected based on the distribution of COVID-19 positive and negative pre-pandemic samples and the assay improved specificity. The horizontal lines indicate the mean values, and the dotted lines represent the cutoff at 30% inhibition. The *P* values presented in (**a**) and (**b**) were calculated from unpaired two-tailed Student’s *t* tests. (**c**) Assay robustness. COVID-19 positive (n = 58) and negative pre-pandemic (n = 101) plasma samples were tested on two different dates. (**d**) COVID-19 positive plasma samples with varying percent neutralization values were titrated to determine the NC_50_ values. (**e**) Correlation analysis of Lumit Spike RBD:ACE2 Immunoassay with an in vitro surrogate viral neutralization assay (sVNT). Serially diluted plasma samples with percent neutralization values ranging from 50 to 90% at 1/20 final plasma dilution were tested with both Lumit and the sVNT (c-Pass), and NC_50_ values were determined and compared. The data presented are the log of the IC_50_ values for sVNT and Lumit and are the mean from two independent experiments. Correlation and linear regression analyses in c and e were performed in GraphPad Prism using Pearson’s correlation coefficients. Statistical significance was calculated using the two-tailed test.

Our earlier results suggested that the in vitro Spike RBD:ACE2 assay could detect the presence of neutralizing antibodies in COVID-19 positive samples. To assess its usefulness as a method for population screening, we further evaluated the Madison patient cohort and the negative pre-pandemic serum samples (true negatives) at a 5% final concentration. The results indicate that the true negative samples clustered at or below 30% neutralization, whereas most true positive samples were distributed along 30–100% neutralization (Fig. 5a).

Defining a threshold value is important for accurately detecting positive and negative samples, while avoiding the occurrence of false positives/negatives. Our results suggest that a cutoff value of 30% would allow the discrimination between the two groups with ~ 80% sensitivity and ~ 100% specificity, therefore avoiding the incorrect calling of false positives (Fig. 5a and Supplementary Fig. 2b, c). We therefore selected a cutoff value of 30% neutralization for subsequent experiments.

We also evaluated plasma samples from a second Madison patient cohort comprising 116 COVID-19 positive and 202 negative pre-pandemic samples at a 5% final concentration. A similar distribution among true negative samples and true positive samples was observed, with both groups clustering below and above the established cutoff value, respectively (Fig. 5b). The sensitivity and specificity values for the plasma samples were 92% and 96%, respectively (Fig. 5b). To ensure assay reproducibility, the plasma samples were evaluated on different dates, and the results show a high correlation among the samples tested, indicating that the Spike RBD:ACE2 immunoassay is robust (Fig. 5c).

Finally, we compared the Spike RBD:ACE2 immunoassay with a similar approach recently developed as a surrogate viral neutralization test that is based on the detection of RBD and ACE2 interaction in a standard ELISA format^14^. To compare the two assays, plasma samples from positive COVID-19 patients that produce percent neutralization values ranging from 50 to 100% in the Lumit Spike RBD:ACE2 immunoassay when used at 1/20 dilution (5% plasma) were selected. Samples were first titrated to determine the possibility of generating NC_50_ values—i.e., dilution factor producing 50% neutralization (Fig. 5d). Then, 15 plasma samples were re-titrated and tested in parallel using both methodologies, and the resulting NC_50_ values were plotted. The results showed a good correlation between both methods (R^2^ = 0.9684) (Fig. 5e). Since the reported assay has been validated against PRNT and VNT^14^, our results suggest that the Spike RBD:ACE2 immunoassay could also be used as a qualitative surrogate viral neutralization test.

**Figure 6:**
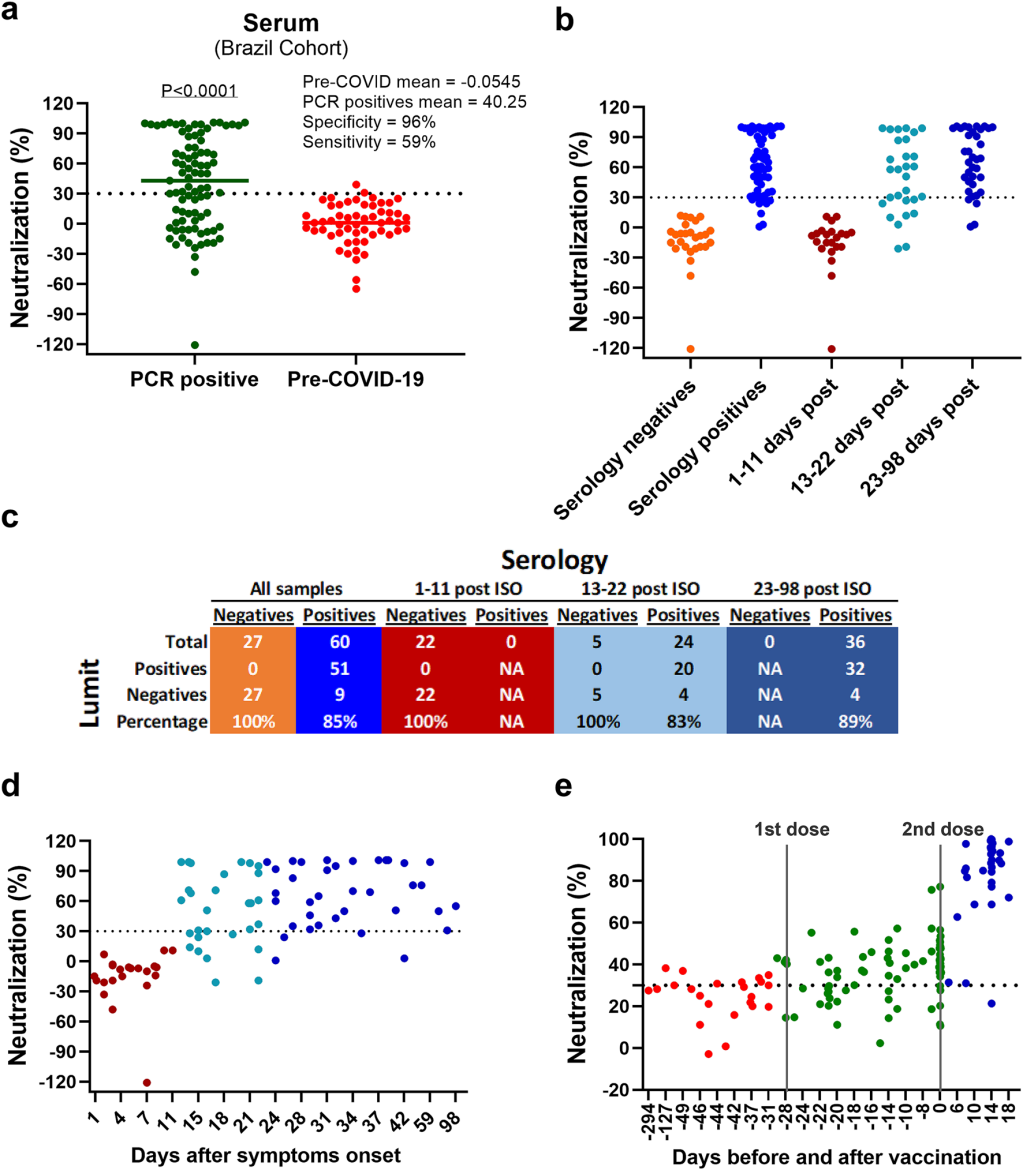
Evaluation of patient-derived samples from a Brazilian cohort. (**a**) COVID-19 positive (n = 87) and negative pre-pandemic (n = 55) samples were tested using the Lumit SARS-CoV-2 Spike RBD:ACE2 Immunoassay at 1/20 (5%) final serum dilution. The sensitivity and specificity values are 59% and 96%, respectively. The horizontal lines indicate the mean values, and the dotted line represents the cutoff at 30% inhibition. The *P* value presented was calculated from unpaired two-tailed Student’s *t *tests. (**b**) Lumit Detection of neutralizing antibodies in PCR-COVID-19 positive samples over time and comparison with results generated by an ELISA-based serological assay (**c**) Percent correlation between Lumit and ELISA-based serological assay data, which targets the trimeric Spike protein (**d**) Antibodies that disrupt the Spike RBD:ACE2 interaction were monitored for at least 90 days after the initial symptom onset in serum of 25 patients in the Brazilian patient cohort. Results are representative of two independent experiments. (**e**) Time-course of neutralizing anti-Spike RBD antibodies in immunized individuals. Individuals immunized with CoronaVac vaccine (n = 28) were tested using Lumit SARS-CoV-2 Spike RBD:ACE2 Immunoassay. Serum samples collected before and throughout the vaccination period were pre-incubated with RBD-rabbit Fc for 30 min prior to the addition of the other assay components. The number of samples tested positive for the presence of neutralizing antibodies increase after the second dose (i.e.: day zero). First and second dose days are represented in vertical lines.

A geographically distinct positive COVID-19 patient cohort from Rio de Janeiro, Brazil was also evaluated with the Spike RBD:ACE2 immunoassay. Eighty-seven positive COVID-19 samples (i.e.: confirmed by either PCR or serological assays) from 25 patients were collected at different time points after the initial symptoms onset (ISO) (i.e.: ranging from day 1 up to day 98 after ISO). For comparison, 55 negative samples collected in Rio de Janeiro prior to the COVID-19 pandemic were also evaluated. A distribution of true negative samples and true positive samples like the Madison cohort was observed, with both groups clustering below and above the established 30% cutoff value, respectively (Fig. 6a). The sensitivity and specificity values observed were 59% and 96%, respectively (Fig. 6a). When the positive COVID-19 samples were evaluated over time, it is noteworthy that samples capable of disrupting the Spike RBD:ACE2 interaction can be detected as early as 13 days after ISO, and the number of positive samples increased over time until 98 days after ISO (Fig. 6b–d and Supplementary Fig. 3). The percentage of true positives correctly identified using the 30% cutoff increase after 23 days after ISO to 89% (Fig. 6c), in close agreement with the sensitivity values observed with the Madison patient cohort samples corresponding to 16–70 days after ISO (Fig. 5b). Also, we observed a good agreement between our results and the total IgG ELISA-based serological assay data, which targets the trimeric Spike protein (Fig. 6c). Finally, we monitored the persistence of circulating anti-SARS-CoV-2 antibodies in samples collected from seven patients for a period of 6 months (Supplementary Fig. 4). Our results indicate that anti-SARS-CoV-2 Spike antibodies capable of disrupting the Spike RBD:ACE2 interaction could be detected in all patients followed, suggesting that humoral immunity may persist for long periods. This observation agrees with recent findings^24^.

### Anti-Spike RBD antibodies are detected in sera from individuals immunized with CoronaVac

Vaccination efforts in Brazil began in late January 2021 when doses of CoronaVac (SinoVac, China), a purified inactivated virus-based vaccine, became available. Although the Brazilian regulatory agency ANVISA approved its use based on clinical trial results, there are very few reports on its efficacy beyond Phase1/2 trials with immunized individuals from ages 18 and older^25,26^. As samples from immunized patients became available, we evaluated the antibody response elicited by the vaccine in individuals that received up to two doses in Rio de Janeiro. Twenty-seven individuals who were not previously exposed to SARS-CoV-2 received two doses of the vaccine 28 days apart on average, and one individual received only one dose at the time of this study. Samples representing different time points (i.e.: before and after vaccine doses) were collected from all individuals.

Spike RBD:ACE2 disrupting antibodies were detected in 27 individuals that received at least one vaccine dose, and percent neutralization values above 60% were observed in 26 individuals after receiving the second dose (Fig. 6e and Supplementary Fig. 5). The presence of neutralizing antibodies could be detected after a single dose, even though the percent neutralization values observed in these samples were lower than from samples collected after the second dose. Also, the detection of RBD:ACE2 disrupting antibodies using the Lumit assay agreed with an in-house ELISA method used to independently characterize these samples (Supplementary Fig. 5). It is noteworthy that the vaccine did not appear to elicit a response in one patient who received two doses (i.e.: patient 16) during the course of this study, as suggested by the negative results observed with both assays (Supplementary Fig. 5) at day 7 after the second dose. However, a delayed immune response to the vaccine is suggested by the positive ELISA result obtained with the last data point at day 14, since the ELISA method detects total IgG against Spike protein. Overall, our results indicate that the Lumit RBD:ACE2 immunoassay could be deployed for high throughput tracing of seroconverted individuals that were either exposed to SARS-CoV-2 or immunized in the ongoing vaccination efforts to assess population immunity.

## Discussion

One year into the pandemic, the world has witnessed an unprecedented deployment of assay methods for COVID-19 detection and diagnosis to trace and contain the spread of this deadly disease. To better understand the epidemiology of this novel coronavirus and to help identify therapies against the COVID-19 disease, several methods were developed for detection of neutralizing antibodies in seroconverted patient samples and for the screening of molecules capable of inhibiting SARS-CoV-2 infection. These assays either detect the effect of neutralizing antibodies on the RBD and ACE2 interaction using ELISA format or use standard virus or viral like particles in PRNT or VNT formats, respectively. Some of the drawbacks of these methods include lengthy protocols, cell engineering, and/or the requirement of high biosafety level work environments. Moreover, many of these assays are not adaptable to high throughput format to screen inhibitor libraries or large patient sample cohorts. We believe the bioluminescent method described here could be deployed for the discovery of novel antiviral drugs, characterizing the role of RBD mutations in viral infectivity, as well as for assessing population immunity to name a few examples.

As a proof-of-concept for drug discovery applications, the Lumit RBD:ACE2 immunoassay enabled us to identify candidate monoclonal antibodies from an assembled mini library of anti-Spike protein antibodies and characterize their potencies, as well as evaluate a small subset of ACE2-derived mini proteins for their inhibition of the RBD:ACE2 PPI (Fig. 3). These results validate the assay as a screening tool and suggest that novel entities interfering with the RBD:ACE2 interaction with potential to become therapies against COVID-19 could be discovered from larger screening libraries. Several groups have reported the development of antibody drugs isolated from genetically humanized mice and from B cells isolated from convalescent patients^10,22,27,28^. The availability of a Spike RBD:ACE2 high throughput screening assay that can be miniaturized while maintaining a stable signal could accelerate the discovery of more therapeutic antibodies.

Our results indicate that an in vitro RBD:ACE2 assay can be used to uncover the contribution of multiple mutations within RBD on the interaction with hACE2. Recently reported variants B.1.1.7 (United Kingdom), B.1.351 (South Africa) and P.1 (Brazil) share the N501Y mutation within Spike RBD, which was recently associated with increased infectivity^20^. Also, neutralization was reduced or abolished when monoclonal antibodies or convalescent sera related to wild type Spike RBD were used^18,19,21,29^. The evaluation of several RBD mutations in a competition assay indicated that two mutations, Y453F and N501Y, increased apparent affinity of RBD to ACE2 eight to tenfold compared to wild type RBD (Fig. 4d).

To evaluate the effect of these mutations on antibody binding and RBD:ACE2 PPI inhibition, we generated RBD-Fc variants containing the mutations Y453F, N501Y and E484K. The mutations E484K and N501Y are found in the B.1.351 (South Africa) and P.1 (Brazil) variants. The Y453F mutation was originally identified in a variant isolated from farmed minks. We found that this mutation increased RBD binding toward ACE2 eight-fold and it did not affect inhibition by the antibodies tested. This was in agreement with the reported data showing that this mutation increases affinity toward ACE2 four-fold although it did not affect neutralization^30^. Our results also indicate that the N501Y mutation affected one antibody binding and decreased its inhibition potency, in agreement with recent observations (Fig. 4e, g). Surprisingly, although the presence of the E484K mutation did not significantly affect the RBD:ACE2 interaction (Fig. 4c), this mutation abolished the PPI inhibition by one antibody evaluated (antibody 5). This suggests that the Glutamic acid E484 of RBD may not be playing an important role in the RBD:ACE2 interaction or its conversion to Lysine did not significantly change the binding interface. However, this amino acid seems to be a critical part of the epitope recognized by antibody 5 and its conversion to Lysine promoted the escape of inhibition by this antibody. We also identified antibodies capable of inhibiting all RBD-Fc variants tested (antibodies 1 and 2). Our results suggest that it might be possible to identify anti-SARS-CoV-2 pan-inhibiting antibodies against the known variants to counteract inhibition escape due to RBD mutations. Alternatively, biotherapies based on antibody combinations may also be an effective approach.

As a method for assessing neutralizing antibody levels in patient-derived samples, the bioluminescent RBD:ACE2 immunoassay readily detected neutralizing activities in positive COVID-19 sera and plasma from seroconverted individuals from two geographically distinct regions (Figs. 5, 6), without the need for viable SARS-CoV-2 virus or pseudotyped viral particles, hence not requiring higher biosafety laboratory requirements for containment of infectious agents (i.e.: BSL-3 for PRNT) or cell culture capabilities (i.e.: BSL-2 for VNT). An in vitro surrogate viral neutralization test (sVNT) that monitors Spike RBD:ACE2 interaction in ELISA format was recently reported to correlate with both PRNT and VNT methods^14^. Although we did not demonstrate correlation between the Lumit Spike RBD:ACE2 immunoassay and either cell-based method, our results showed a high correlation with the reported sVNT assay (Fig. 5e). Since our method is amenable to high throughput testing, we believe it could be readily deployed for screening of a large patient population for epidemiological studies as well as to assess the success of vaccination efforts—i.e., population immunity.

Our results suggest that a humoral response against SARS-CoV-2 may last longer than 6 months (Fig. 6d and Supplementary Fig. 4), in agreement with recent reports^31–33^. This finding could have several implications. First, it suggests that protective immunity may persist in individuals exposed to SARS-CoV-2 for at least 6 months. The second implication relates to vaccination response. The results presented here from a limited number of vaccinated patients suggest that the vast majority developed neutralizing antibodies, and the results were corroborated with serological ELISA assay, which detects antibodies against the trimeric SARS-CoV-2 Spike protein. Follow-up of a larger cohort of CoronaVac-immunized individuals is warranted to determine the longevity of the antibody response and the reduction on the number of infections, but these preliminary results are promising and reinforce the necessity of deploying all available resources to contain the spread of SARS-CoV-2. Recent reports on the reduction of infections among healthcare workers from the United States and Israel immunized with mRNA vaccines are encouraging signs of the effectiveness of the current vaccination campaigns^34–37^. It is noteworthy that individuals immunized with the BNT162b2 mRNA vaccine who were recently infected with SARS-CoV-2 elicited higher levels of neutralizing antibodies compared to those without an infection history^35,38^. We also observed a similar pattern in individuals who were infected with SARS-CoV-2 prior to immunization with CoronaVac (O. C. Ferreira, unpublished). Overall, these observations suggest that a single vaccine regimen would be sufficiently protective in these individuals, which could help alleviate the current vaccine shortage by making more doses available for individuals in higher risk groups.

In summary, our results indicate that a homogeneous bioluminescent Spike RBD:ACE2 immunoassay could have a broad utility, from enabling the screening of novel viral entry inhibitors, to monitoring the efficacy of newly developed antibodies against the virus, and the detection of patient-derived circulating antibodies.

## Methods

### Reagents

The following proteins were purchased from Sino Biological (Beijing, China): SARS-CoV-2 recombinant Spike RBD wild type, RBD (S477N), RBD (Y453F), RBD (K458R), RBD (F342L), RBD (V367F), RBD (N354D), RBD (A435S), RBD (V483A), RBD (W436R), RBD (G476S), RBD (R408I), RBD (K417N), RBD (Y505C), RBD (N501Y), and human ACE2-histag. The different anti-SARS-CoV-2 Spike protein antibodies were purchased from Sino Biological, Active Motif (Carlsbad, USA), Biolegend (San Diego, CA), ACRO Biosystems (Newark, USA), and Absolute Antibody (Oxford, United Kingdom) (Table 1). HRP conjugated anti-human IgG was from Southern Biotech (Birmingham, AL, USA). Human ACE2-derived peptides described by Cao et al.^9^ were custom ordered from Peptide 2.0 and the list is presented in Supplementary Table 1.

Lumit SARS-CoV-2 Spike RBD:ACE2 immunoassay components were from Promega (Madison, USA) and they consist of 0.5 μM SARS-CoV-2 RBD-rabbit Fc (RBD-Fc), 0.5 μM human ACE2-mouse Fc (ACE2-Fc), Lumit Detection Substrate C, 10X Lumit Immunoassay Buffer C, Lumit anti-rabbit Ab-SmBiT and Lumit anti-mouse Ab-LgBiT. The Lumit anti-rabbit Ab-LgBiT and Lumit anti-mouse Ab-SmBiT used in some control experiments were also from Promega. c-Pass kit was purchased from Genscript (Piscataway, New Jersey).

### Human derived samples used in this study

COVID-19 positive and negative samples (serum and plasma) were obtained from individuals at the Promega Madison on-campus clinic (USA). The samples were collected with written informed consent and approved by the Human Subjects Board (HSB Protocol #21). For Brazil study, the sera were collected at the Federal University of Rio de Janeiro (Brazil), and consent was obtained prior to sample collection. The study was approved by the local ethics committee from Clementino Fraga Filho University Hospital (CAAE: 30161620.0.0000.5257) and national ethical review board (CAAE 30127020.0.0000.0068). All methods were performed in accordance with the relevant guidelines and regulations and informed consent was obtained from all subjects. Additional COVID-19 confirmed negative serum samples were obtained from Lee BioSolutions (Maryland Heights, USA).

### Lumit SARS-CoV-2 Spike RBD:ACE2 immunoassay protocol

Except where indicated, the general protocol of the PPI immunoassay in 96 well plates used a final concentration of 1.5 nM RBD-Fc and 1.5 nM ACE2-Fc, and the assay reaction consists of adding 5 µl of patient-derived samples or inhibitor diluted in 1× Lumit immunoassay buffer, to 10 µl of 7.5 nM RBD-rabbit Fc and 10 µl of 7.5 nM human ACE2-mouse Fc. Then, 25 µl of Lumit Antibody Mix composed of Lumit anti-rabbit Ab-SmBiT and Lumit anti-mouse Ab-LgBiT (0.3 µl each), diluted in 1× Lumit immunoassay buffer were added. The plate is incubated at room temperature (~ 23 °C) for 60 min, followed by the addition of 12.5 µl of Lumit Detection Reagent (Lumit substrate mixed with 1× Lumit immunoassay buffer) to the reactions and incubated for 30 min at room temperature. Luminescence was measured using a plate-reading luminometer (Fig. 1a).

### SARS-CoV-2 Spike RBD and human ACE2 protein–protein interaction assay optimization

Serial dilutions of RBD-rabbit Fc and ACE2-mouse Fc were used to determine the linearity and sensitivity of the detection of protein–protein interaction using Lumit methodology. RBD-Fc and ACE2-Fc proteins were diluted in 1× Lumit immunoassay buffer to 10 nM of each of the proteins. The 10 nM RBD:ACE2 solution was serially diluted in 12 wells of a 96-well plate to produce a final dilution series from 2 to 0.2 nM, plus a 0 nM protein sample—i.e., Lumit antibodies-only control. Ten microliters of each dilution were transferred to a 96-well assay plate, followed by 5 µl of 1× Lumit immunoassay buffer and Lumit Antibody Mix. The Spike RBD:ACE2 PPI was detected as described in the general protocol above. Luminescence signal stability after addition of Lumit Detection Reagent was evaluated using 1.5 nM of each of RBD-Fc and ACE2-Fc proteins for 120 min after the initial luminescence read at the 30-min time point.

### Anti SARS-CoV 2 Spike antibody screening and characterization

Antibody screening experiments were performed in 96-well plates at room temperature. Chimeric or patient-derived monoclonal anti-SARS-CoV-2 Spike protein antibody working solutions were prepared in 1× Lumit immunoassay reaction buffer and evaluated at a final concentration of 55 nM in a 50 µl-reaction containing 1.5 nM RBD-Fc, 1.5 nM ACE2-Fc, and Lumit antibodies in 1× Lumit immunoassay reaction buffer. The reaction steps were as follows: first, the anti-SARS-CoV-2 Spike protein antibodies were incubated for 30 min with RBD-Fc. Then, the remaining components of the reaction were added, and the incubation was continued for another 60 min at 23 °C, followed by addition of Lumit Detection Reagent. Luminescence was read after 30-min incubation.

Primary hits from the antibody screening were selected for follow-up characterization. Antibody working solutions were four-fold diluted from 500 to 0.002 nM final in a 50 µl-reaction containing 1.5 nM RBD-Fc, 1.5 nM ACE2-Fc, and Lumit antibodies in 1× Lumit immunoassay reaction buffer. The reaction steps were as follows: first, the antibody dilutions were incubated for 30 min with RBD-Fc. Then, the remaining components of the reaction were added, followed by 60-min incubation at 23 °C, and addition of Lumit Detection Reagent. Luminescence was read after a 30-min incubation. Antibodies were tested to assess possible interference with the bioluminescence signal. Antibody dilutions were incubated with a luminescence-generating reaction that contained either 1.5 nM RBD-rabbit Fc and Lumit Anti-Rabbit (SmBit/LgBit) antibodies, or 1.5 nM ACE2-mouse Fc and Lumit Anti-Mouse (SmBit/LgBit) antibodies in 1× Lumit immunoassay reaction buffer, and assay was performed as described above.

### Peptide inhibitor characterization

Inhibitory peptide working solutions were four-fold diluted from 2000 to 0.002 nM or from 10 to 0 µM in a 50 µl-reaction containing 1.5 nM RBD-Fc, 1.5 nM ACE2-Fc, and Lumit antibodies in 1× Lumit immunoassay reaction buffer. After a 30-min incubation of the antibody dilutions with RBD-Fc, the remaining components of the reaction (ACE2-Fc, Lumit antibodies) were added and the reactions were incubated for 60 min at 23 °C, followed by addition of Lumit Detection Reagent. Luminescence was read after 30-min incubation. Peptides were also tested to assess potential assay interference. Serially diluted peptides were incubated with either 1.5 nM RBD-Fc and Lumit Anti-Rabbit (SmBit/LgBit) antibodies, or 1.5 nM ACE2-Fc and Lumit Anti-Mouse (SmBit/LgBit) antibodies in 1× Lumit immunoassay reaction buffer, and assay was performed as described above.

### Characterization of patient-derived samples for neutralizing effect

Negative pre-pandemic serum was evaluated at different concentrations to determine the matrix effect on Lumit SARS-CoV-2 Spike RBD:ACE2 Immunoassay performance. Serum was diluted from 1/1280 to 1/2.5 final dilution factor in 1× Lumit immunoassay reaction buffer and used in the Lumit SARS-CoV-2 Spike RBD:ACE2 immunoassay. The 1/20 dilution factor (5%) serum was selected for subsequent experiments.

Negative pre-pandemic and patient-derived COVID-19 PCR positive samples were prepared as follows. 50% patient-derived sample solutions (serum and plasma) were prepared using 1× Lumit immunoassay reaction buffer. Five microliters of the diluted samples were transferred to a 96-well assay plate. Then, 10 µl of 7.5 nM RBD-Fc was added to the assay plate wells and the mixtures were incubated at 23 °C for 30 min, followed by the addition of 10 µl of 7.5 nM ACE2-Fc and 25 µl of Lumit Antibody Mix. After 60 min incubation at 23 °C, 12.5 µl of Lumit Detection Reagent was added to the wells, and the plate was incubated for another 30 min before luminescence was recorded. In addition to multiple known negative pre-pandemic samples that will generate the highest light output, a “Lumit antibodies-only” control is added to the plate for the lowest light output. This control sample where RBD and ACE2 proteins were omitted contains 5% negative serum and Lumit Antibody Mix in 50 µl reaction.

### ELISA anti-Spike IgG assay

Microtitre plates (Immulon 2 HB) were coated with trimeric spike proteins (4 μg/ml), incubated overnight at 4 °C, and blocked with 5% BSA in PBS + 0.05% Tween-20 (PBST). Samples diluted at 1/50 in PBST + 2% BSA were added and the plates were incubated 1 h at 37 °C. After washing, HRP conjugated anti-human IgG were added. After 1 h at room temperature, reactions were developed with TMB, stopped with 1 N sulphuric acid, and read at 450 nm (Biochrom Asys reader). Samples from a COVID-19 positive case and from a pre-pandemic period (in triplicate) were added as control and for cut-off determination. Results were expressed as reaction of sample optical density value divided by assay cutoff (S/C ratio). An S/C ratio < 1 is negative; > 1.5 is positive and borderline if between these values.

### Signal detection and data analysis

All 96-well, 384-well and 384-well low volume assay plates were read using the GloMax Discover Luminometer from Promega. The instrument was set to 0.5 s integration time. Data was analyzed using Microsoft Excel and GraphPad Prism, version 8 software. IC_50_ values were determined by using a nonlinear regression fit to a sigmoidal dose–response (variable slope).

To analyze data from the patient-derived samples and identify samples positive for NAbs, relative luminescence units (RLUs) were converted to net luminescence values by subtracting the “Lumit antibodies-only” control RLU values from luminescence values generated by the negative pre-pandemic and patient-derived COVID-19 PCR positive samples. Then, the net luminescence values of all samples were converted to percent PPI activity using the “Lumit antibodies-only” and the average “Pre-COVID-19 negative serum” controls as 0 and 100%, respectively. The % neutralization or inhibition is calculated using the following formula

$${\text{Sample }}\% {\text{ Neutralization }} = { 1}00 \, - \, \% {\text{ PPI activity}}.$$

By testing multiple PCR negative samples, we established a cutoff of 30% neutralization that should be sufficient to indicate if a sample is positive for NAbs.

## Supplementary Information

Supplementary Information.
