# Supplementary material for: A bioluminescent and homogeneous SARS-CoV-2 spike RBD and hACE2 interaction assay for antiviral screening and monitoring patient neutralizing antibody levels
Source: Sci Rep. 2021 Sep 16;11:18428. doi: 10.1038/s41598-021-97330-3 (PMC8445915; doi:10.1038/s41598-021-97330-3)
Supplement: Supplementary file 1 — Supplementary Information. [file 41598_2021_97330_MOESM1_ESM.pdf]

Supplementary Material

A bioluminescent and homogeneous SARS-CoV-2 spike RBD and hACE2 interaction assay for antiviral screening and monitoring patient neutralizing antibody levels

Juliano Alves[1*], Laurie Engel[1], Renata de Vasconcelos Cabral[2], Eduardo L. Rodrigues[3], Liane de Jesus Ribeiro[2], Luiza M. Higa[2], Orlando da Costa Ferreira Júnior[2], Terezinha Marta P. P. Castiñeiras[4], Isabela de Carvalho Leitão[5], Amilcar Tanuri[2], Said A. Goueli[1,6], and Hicham Zegzouti[1*]

[1] Promega Corporation, R&D Department, Madison, WI, USA

[2] Laboratório de Virologia Molecular, Departamento de Genética, Instituto de Biologia, Universidade Federal do Rio de Janeiro, Rio de Janeiro, Brazil

[3] Promega Biotecnologia do Brasil, São Paulo, Brazil

[4] Departamento de Doenças Infecciosas e Parasitárias, Faculdade de Medicina, Universidade Federal do Rio de Janeiro, Rio de Janeiro, Brazil

[5] Instituto de Biofísica Carlos Chagas Filho, Universidade Federal do Rio de Janeiro, Rio de Janeiro, Brazil

[6] Department of Pathology and Laboratory Medicine, University of Wisconsin School of Medicine and Public Health, Madison, WI. USA

Correspondence and requests for materials should be addressed to:

*Hicham Zegzouti, Ph.D. e-mail: hicham.zegzouti@promega.com

*Juliano Alves, Ph.D. email: juliano.alves@promega.com

a

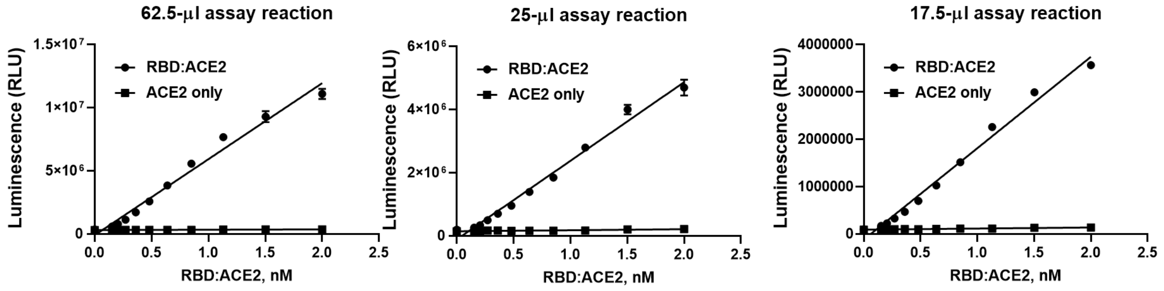

b

	SARS-CoV-2 Spike RBD:ACE2 standard curve - 96-well plate										
RBD:ACE2 (nM)	2.00	1.50	1.13	0.85	0.64	0.48	0.36	0.27	0.20	0.15	0
62.5-µl reaction	11,086,666.7	9,284,333.3	7,662,000.0	5,557,333.3	3,831,000.0	2,572,666.7	1,707,666.7	1,113,333.3	780,966.7	586,233.3	347,700.0
S/B values	32	27	22	16	11	7	5	3	2	2	1

	SARS-CoV-2 Spike RBD:ACE2 standard curve - 384-well plate										
RBD:ACE2 (nM)	2.00	1.50	1.13	0.85	0.64	0.48	0.36	0.27	0.20	0.15	0
25-µl reaction	4,690,750.0	3,997,500.0	2,792,000.0	1,840,000.0	1,389,250.0	952,300.0	699,150.0	492,225.0	333,150.0	255,575.0	150,200.0
S/B values	31	27	19	12	9	6	5	3	2	2	1

	SARS-CoV-2 Spike RBD:ACE2 standard curve - 384-well low volume plate										
RBD:ACE2 (nM)	2.00	1.50	1.13	0.85	0.64	0.48	0.36	0.27	0.20	0.15	0
17.5-µl reaction	3,562,000.0	2,988,000.0	2,256,500.0	1,512,250.0	1,020,000.0	695,450.0	465,850.0	326,075.0	223,250.0	168,700.0	94,652.5
S/B values	38	32	24	16	11	7	5	3	2	2	1

c

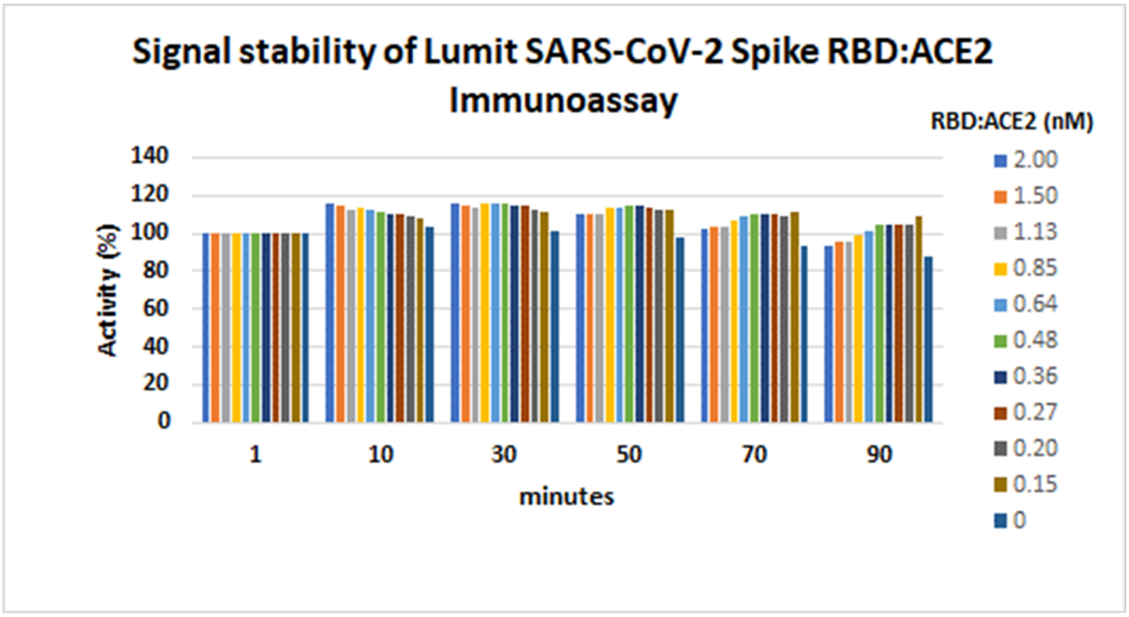

Supplementary Fig. 1: Lumit SARS-CoV-2 Spike RBD: ACE2 Immunoassay features. (a) SARS-CoV-2 Spike RBD: ACE2 linearity curves and determination of the limit of detection using different reaction volumes and plate formats (96-, 384- and 384-well low volume plates). SARS-CoV-2 Spike RBD: ACE2 titration was performed in 1X Buffer C. (b) Signal-to-background values for each SARS-CoV-2 Spike RBD: ACE2 titration are shown. (c) Signal stability as percent change from a 30-minute read after the addition of Lumit Detection Reagent. Results are presented as means ± S.E.M. (n=3).

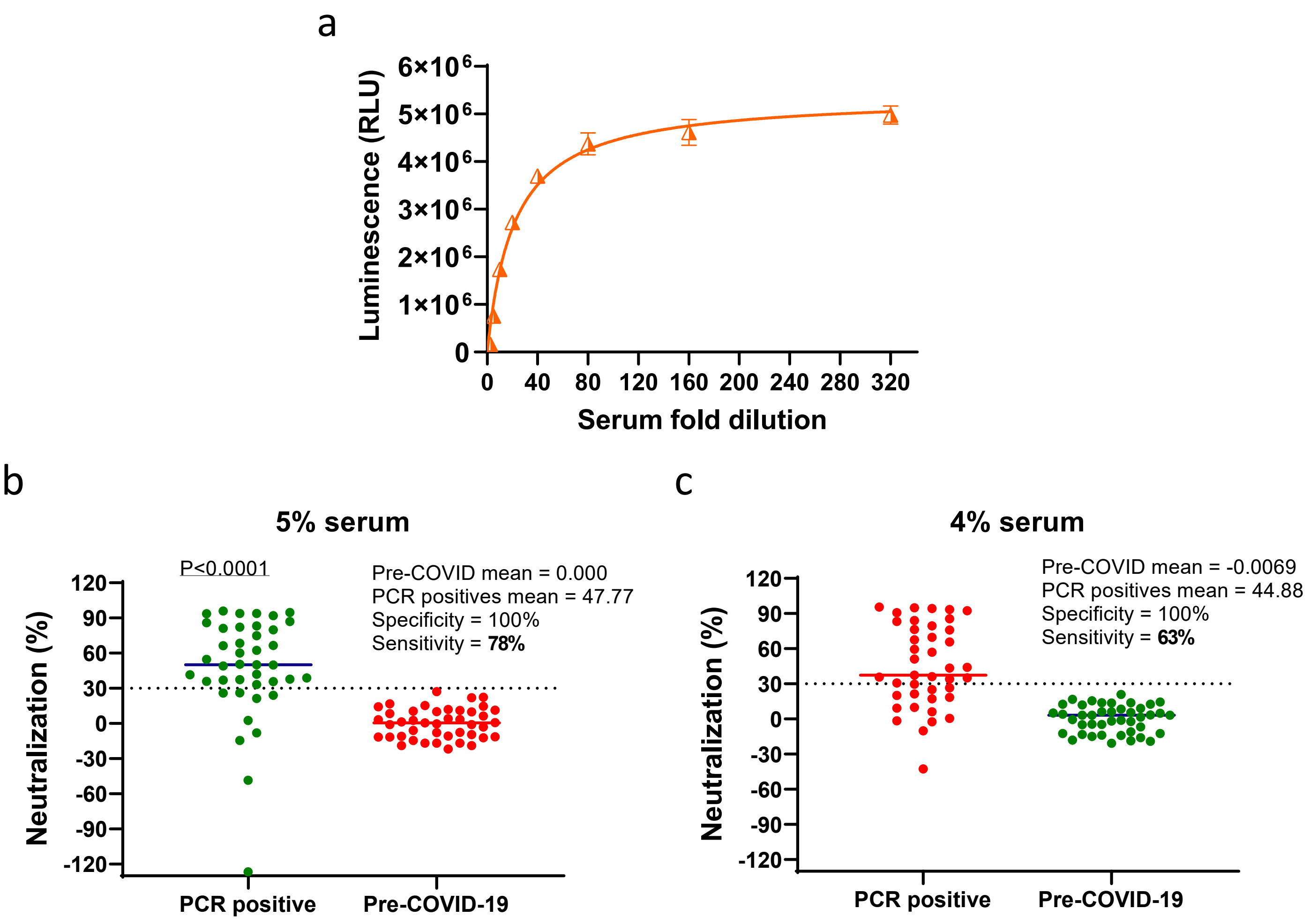

Supplementary Fig. 2. Optimization of Lumit SARS-CoV-2 Spike RBD: ACE2 Immunoassay in patient-derived samples. (a) Effect of serum dilutions on light output generated by Lumit immunoassay. (b-c) Comparison of sensitivity and specificity of the Lumit assay in two different serum dilutions. COVID-19 positive (n=41) and negative pre-pandemic samples (n=43) were tested using Lumit SARS-CoV-2 Spike RBD: ACE2 Immunoassay at 5% and 4% serum. Serum samples were pre-incubated with Spike RBD-rabbit Fc for 30 minutes prior to the addition of the other assay components. A cutoff value of 30% was selected based on the distribution of COVID-19 positive and negative pre-pandemic samples and the assay improved specificity. The horizontal lines indicate the mean values, and the dotted lines represent the cutoff at 30% inhibition. The P values presented in a and b were calculated from unpaired two-tailed Student’s t-tests. Results are representative of two independent experiments

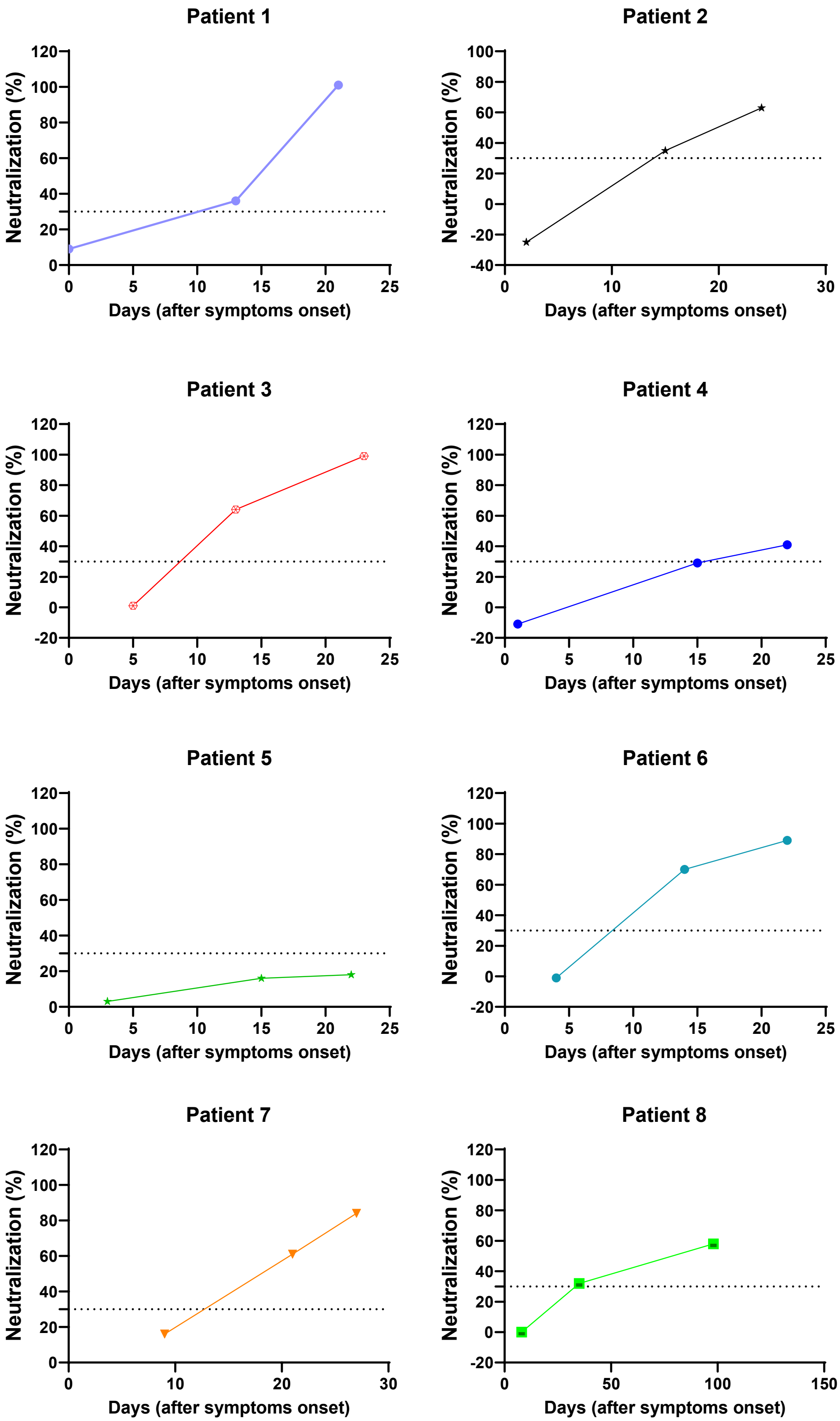

Supplementary Fig. 3. Time-course of patient-derived samples that were collected in separate dates after the initial symptom onset.

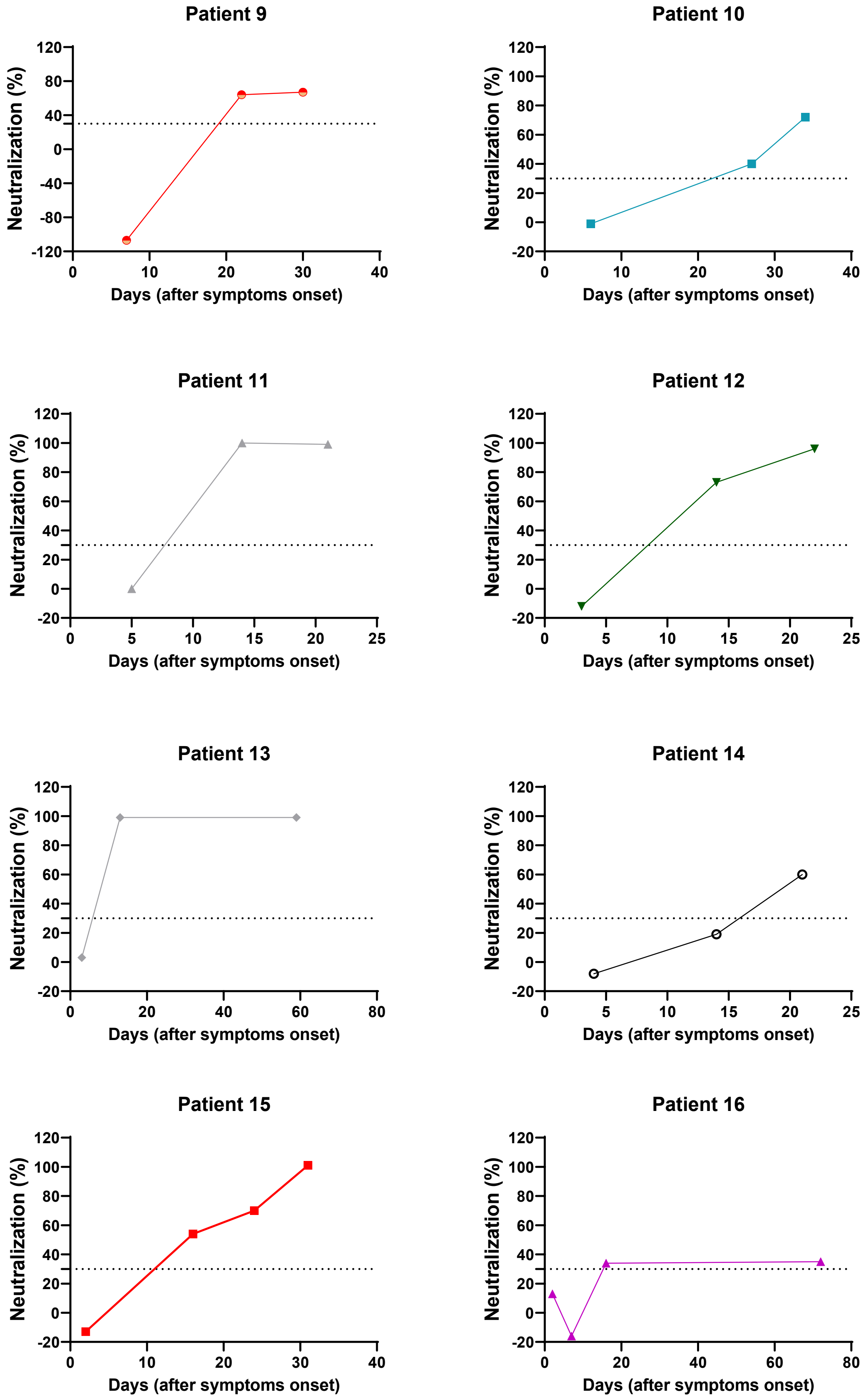

Supplementary Fig. 3 continued

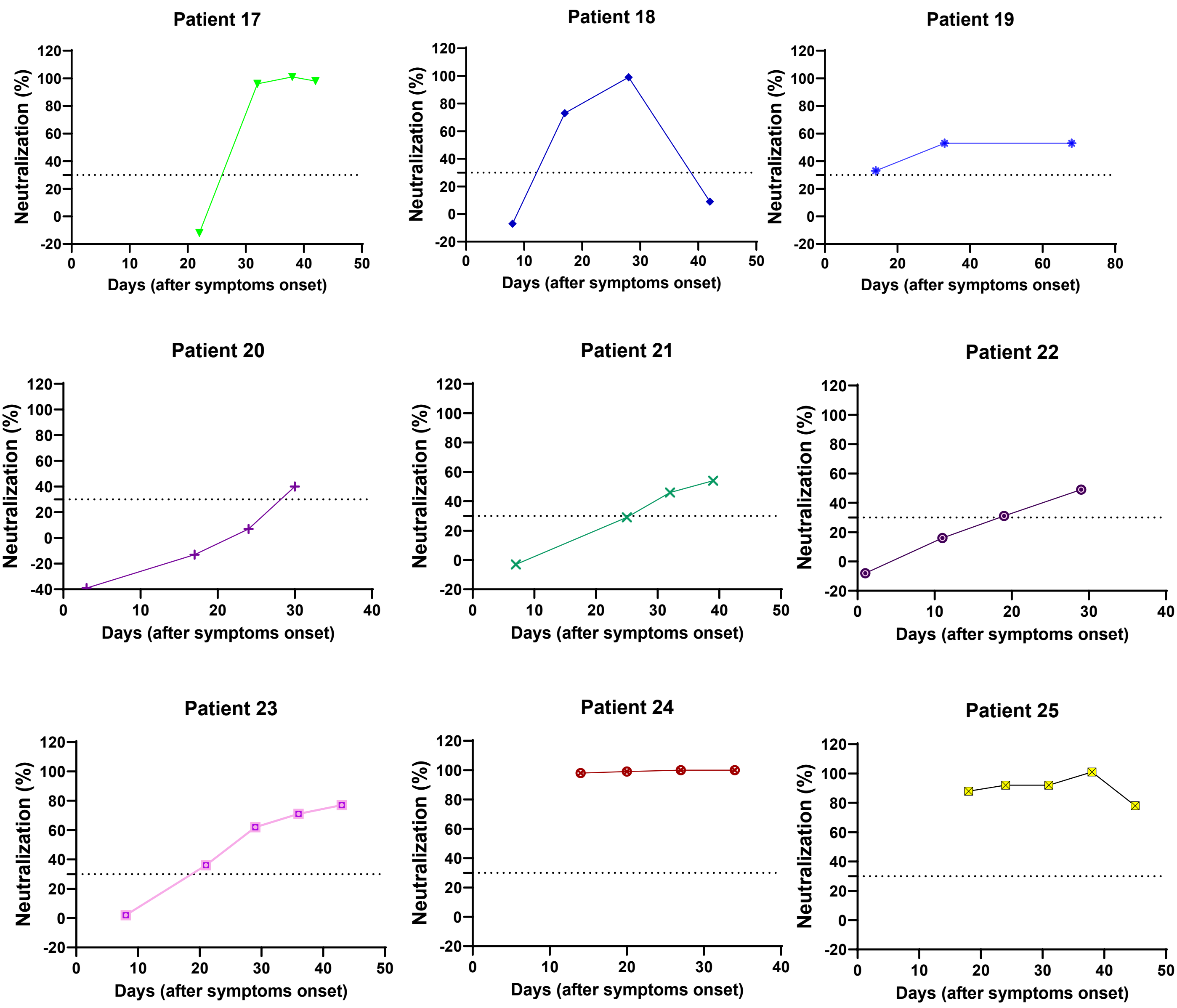

Supplementary Fig. 3 continued

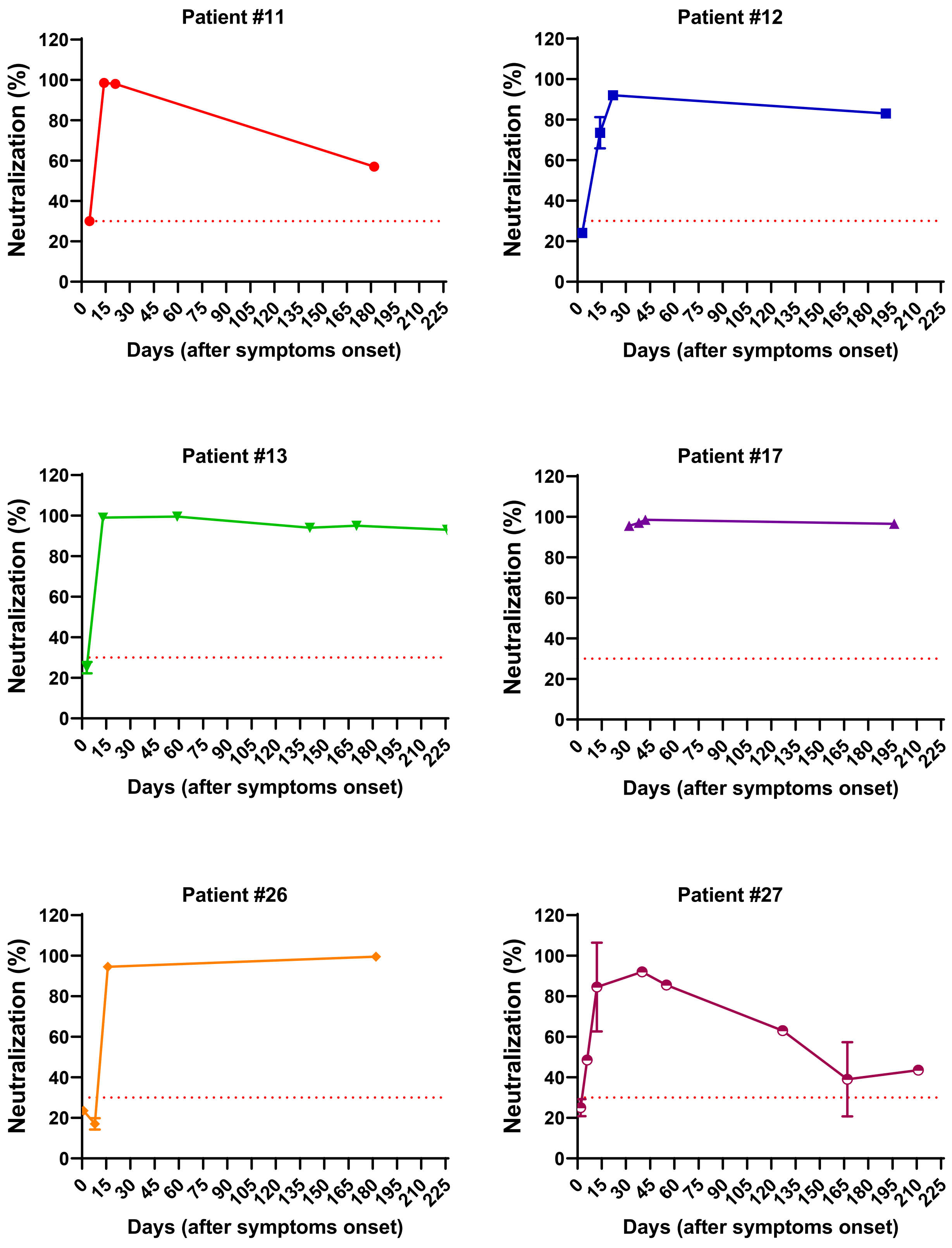

Supplementary Fig. 4. Longer time-course of patient-derived samples that were collected in separate dates after the initial symptom onset and beyond 6 months.

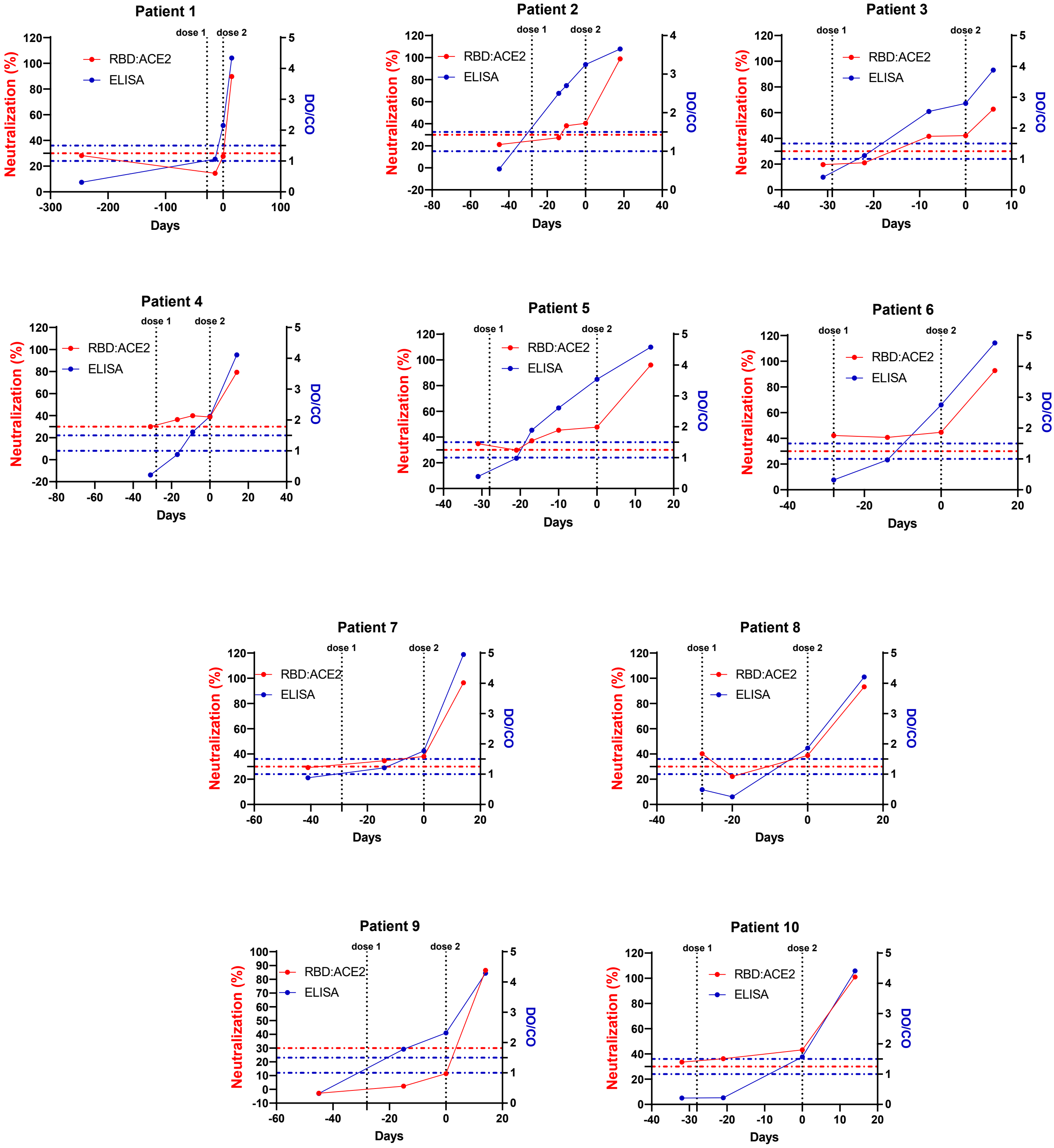

Supplementary Fig. 5. Time-course of patient-derived samples that were collected in separate dates before and after CoronaVac vaccination. Anti-Spike antibodies were detected using Lumit Spike RBD: ACE2 immunoassay and ELISA.

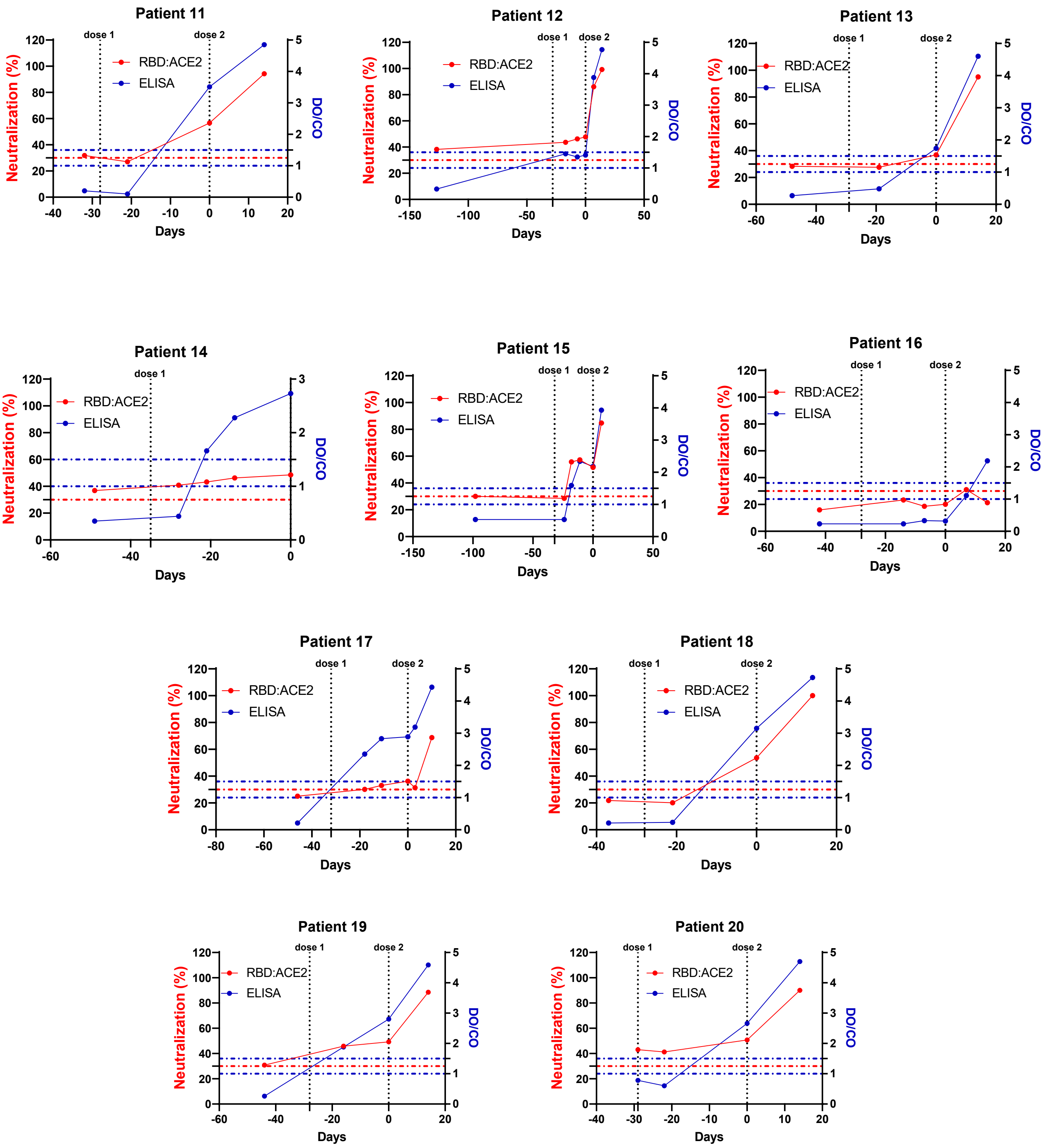

Supplementary Fig. 5 continued

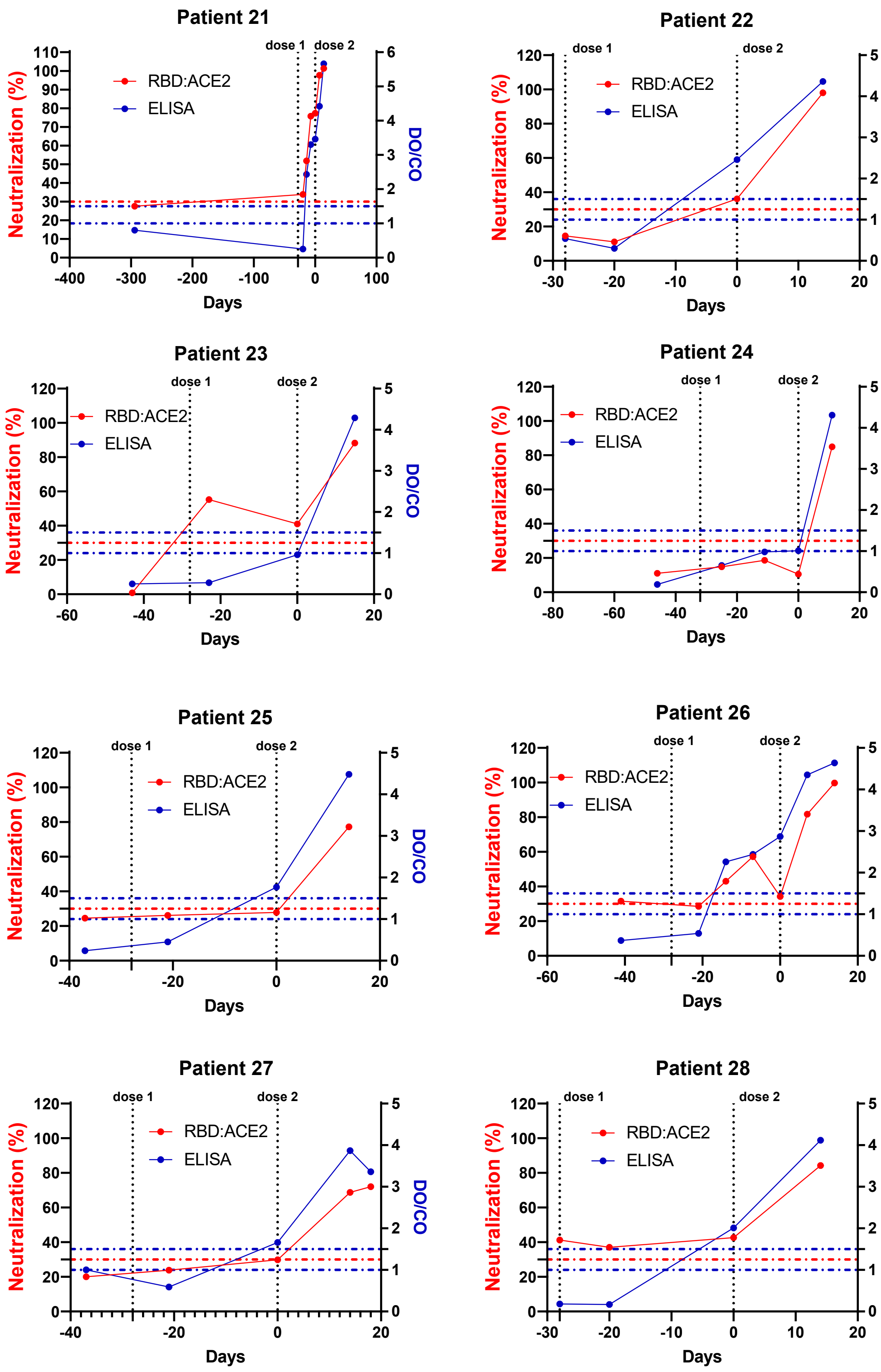

Supplementary Fig. 5 continued

Supplementary Table 1: List of In silico-derived peptide inhibitors[9] used in this study

Name	Peptide Sequence
AHB1	DEDLEELERLYRKAEEVAKEAKDASRRGDDERAKEQMERAMRLFDQVFELAQELQEKQTDGNRQKATHLDKAVKEAADELYQRVR
AHB2	ELEEQVMHVLDQVSELAHELLHKLTGEELERAAYFNWWATEMMLELIKSDDEREIREIEEEARRILEHLEELARK
LCB2	SDDEDSVRYLLYMAELRYEQGNPEKAKKILEMAEFIAKRNNNEELERLVREVKKRL
LCB3	NDDELHMLMTDLVYEALHFAKDEEIKKRVFQLFELADKAYKNNDRQKLEKVVEELKELLERLLS
LCB4	QREKRLKQLEMLLEYAIERNDPYLMFDVAVEMLRLAEENNDERIIERAKRILEEYE
LCB6	DREQRLVRFLVRLASKFNLSPEQILQLFEVLEELLERGVSEEEIRKQLEEVAKELG

Supplementary Table 2: Spike RBD mutants tested in the competition assay and their IC_{50} values

Spike RBD	IC_{50} (nM)	Fold Change in affinity	Country of first report
**K417N**	94.84	5.7 (Decrease)	
**G476S**	38.88	2.4 (Decrease)	USA
**Q414E**	28.08	1.7 (Decrease)	
**E484K**	27.64	1.7 (Decrease)	
**W436R**	21.35	1.3 (Decrease)	Wuhan
**L452R**	20.37	1.2 (Decrease)	
**F342L**	20.35	1.2 (Decrease)	England
**P384L**	18.27	1	
**K417N, E484K, N501Y**	18.08	1	South Africa
**T478I**	17.64	1	
**R408I**	16.76	1	India
**Wild Type**	16.51	1	
**A435S**	15.75	1	Finland
**K458R**	15.54	1	
**N354D**	13.54	1.2 (Increase)	China
**Q414R**	12.05	1.4 (Increase)	
**V483A**	11.95	1.4 (Increase)	USA
**V367F**	11.54	1.4 (Increase)	Hong Kong/ France
**V347F**	11.51	1.4 (Increase)	
**V341I**	9.843	1.7 (Increase)	Wales
**Y508H**	6.865	2.4 (Increase)	
**N439K**	5.658	2.9 (Increase)	
**S477N**	4.62	3.6 (Increase)	Australia
**Y453F**	2.009	8.2 (Increase)	Denmark mink
**N501Y**	1.659	10.0 (Increase)	UK/South Africa/Brazil
